# Site matters: site-specific factors control phosphorus retention in buffer strip soils under concentrated field runoff

**DOI:** 10.1007/s11356-024-34383-7

**Published:** 2024-07-17

**Authors:** David Ramler, Peter Strauss

**Affiliations:** Institute for Land and Water Management Research, Federal Agency for Water Management, Pollnbergstraße 1, 3252 Petzenkirchen, Austria

**Keywords:** Vegetated filter strips, Sediment and nutrient retention, Flow convergence, Concentrated flow, Erosion, Degree of phosphorus saturation (DPS), Phosphorus sorption index (PSI)

## Abstract

Soil erosion from agricultural fields is a persistent ecological problem, potentially leading to eutrophication of aquatic habitats in the catchment area. Often used and recommended mitigation measures are vegetated filter strips (VFS) as buffer zones between arable land and water bodies. However, if they are designed and managed poorly, nutrients — especially phosphorus (P) — may accumulate in the soil. Ultimately, VFS can switch from being a nutrient sink to a source. This problem is further aggravated if the field runoff does not occur as uniform sheet flow, but rather in concentrated form, as is usually the case. To assess the impact of concentrated flow on VFS performance, we have taken soil core samples from field-VFS transition zones at six sites in Lower Austria. We determined a multitude of physical and chemical soil parameters, focusing on P fractions and indices. Our results revealed that concentrated flow can lead to an accumulation of P in the VFS. P levels in the VFS inside the area of concentrated runoff can be equal to or higher than in the field, even though they receive no direct fertilization. However, the concentration and distribution of nutrients in the fields and VFSs were also site-specific and affected by local factors such as the age of the VFS, cropping, and fertilization. Accordingly, there is a need for more sophisticated, bespoke VFS designs that can cope with site-specific runoff volumes and movements of nutrients that occur.

**Supplementary Information:**

The online version contains supplementary material available at 10.1007/s11356-024-34383-7.

## Introduction

Soil erosion and runoff from agricultural areas are still major environmental issues. The nutrients that are transported with the eroded sediment and runoff water in particulate or dissolved form can lead to eutrophication processes in adjacent water bodies (Correll 1998). Phosphorus (P) is of particular concern, as it is commonly the limiting factor in aquatic systems (Ulén et al. 2007; Schindler et al. 2016). Contrary to the more mobile nitrogen, P may accumulate in soils, leading to problems with legacy P stocks, even if fertilization or agricultural use has ceased (Jarvie et al. 2013; Sharpley et al. 2013). Vegetated filter strips (VFS; also termed buffer strips) are often used and recommended measures against erosion and nutrient export. These buffer zones between arable land and water bodies are supposed to slow down the field runoff, thereby promoting infiltration and deposition of transported sediment (Stutter et al. 2012; Prosser et al. 2020). VFSs are considered easy to implement, but several requirements are crucial for proper functioning and effectiveness. These are, however, usually not or only insufficiently considered during VFS design (Ramler et al. 2022).

The full potential for filtering and retention is only realized if the field runoff occurs as uniform sheet flow and is, thus, spread evenly across the whole buffer width. Consequently, concentrated runoff, caused by flow convergence in the field (e.g., due to gully erosion, thalwegs, topography) or at the field edge (e.g., due to barriers, ditches, berms), severely diminishes VFS performance. In this case, only a fraction of the VFS area remains functional but has to deal with the whole field runoff (Dosskey et al. 2002; Pankau et al. 2012). A runoff concentration also implies a higher flow velocity and erosive force, meaning the VFS has less time and space to handle incoming runoff with a higher sediment load. Such flow concentrations are, in fact, frequently encountered and may be considered the dominant type of runoff (Helmers et al. 2005; Hancock et al. 2015; Shrestha et al. 2018).

Furthermore, if VFSs are not designed and managed properly, P and other elements may accumulate in the soil. The long-term P input to the soil must be in balance with the P that can be removed by harvesting the vegetation — otherwise, the VFS has an expiration date (Ramler et al. 2022). Significant amounts of P may already leach from soils even if they are not (yet) saturated (Djodjic et al. 2004; Stutter et al. 2009). VFSs will eventually switch from net nutrient sinks to sources if inputs are higher than outputs.

In this study, we assessed the effect of concentrated flow on buffer strip soils, by taking soil core samples along transects at field-VFS transitions. Specifically, we hypothesized that P concentrations in the soil would be higher within the area of concentrated runoff compared to outside, higher in the field compared to the VFS, and higher at the surface compared to deeper soil layers. Consequently, we expect increased P concentrations in VFS soils within the concentrated runoff area and at subsurface layers. Furthermore, we analyzed a multitude of other physical and chemical parameters, including nutrient pools and P indices.

## Materials and methods

### Experimental setup

**Fig. 1:**
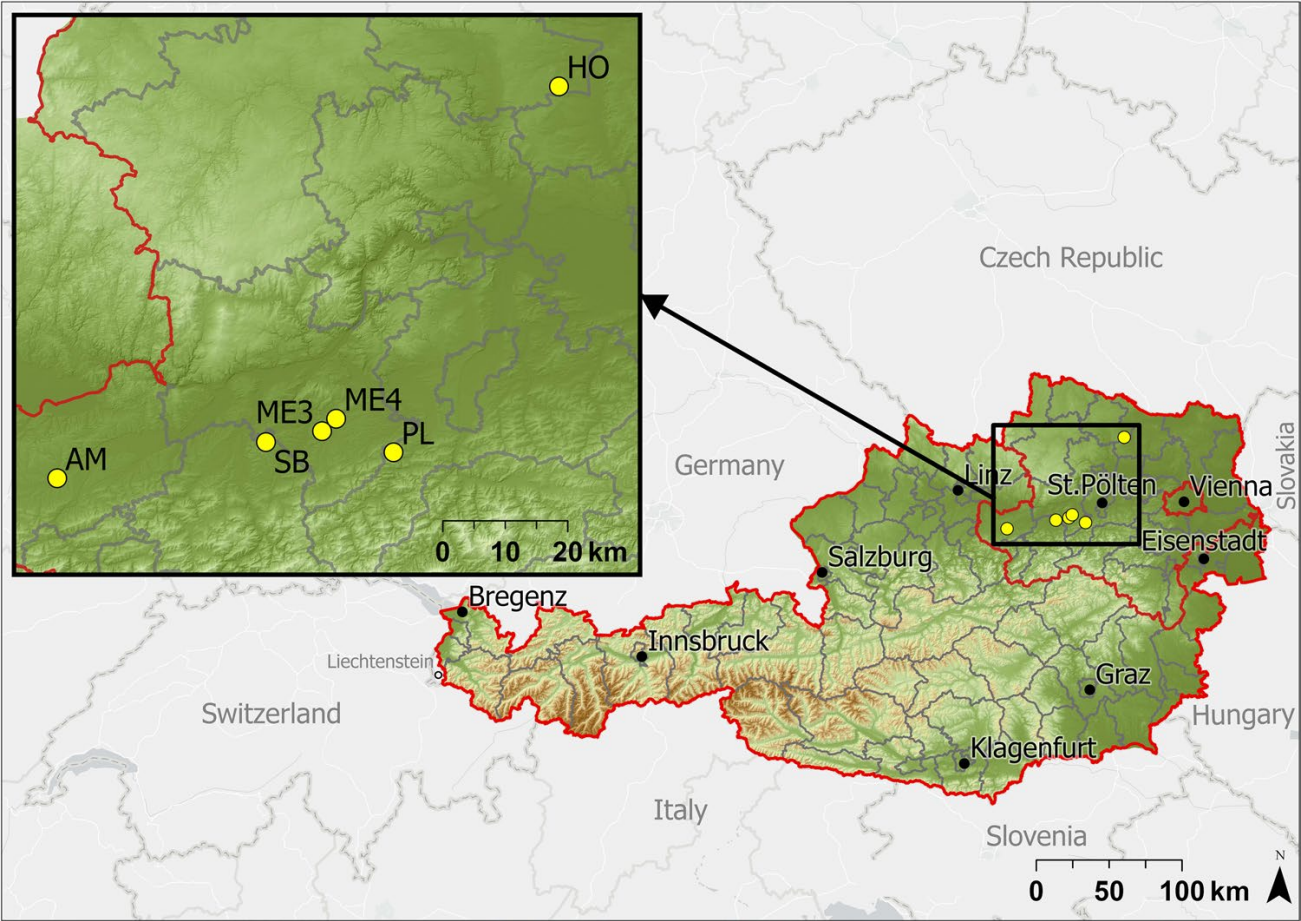
Location of sampling sites in Lower Austria. The gray lines indicate district borders

**Fig. 2:**
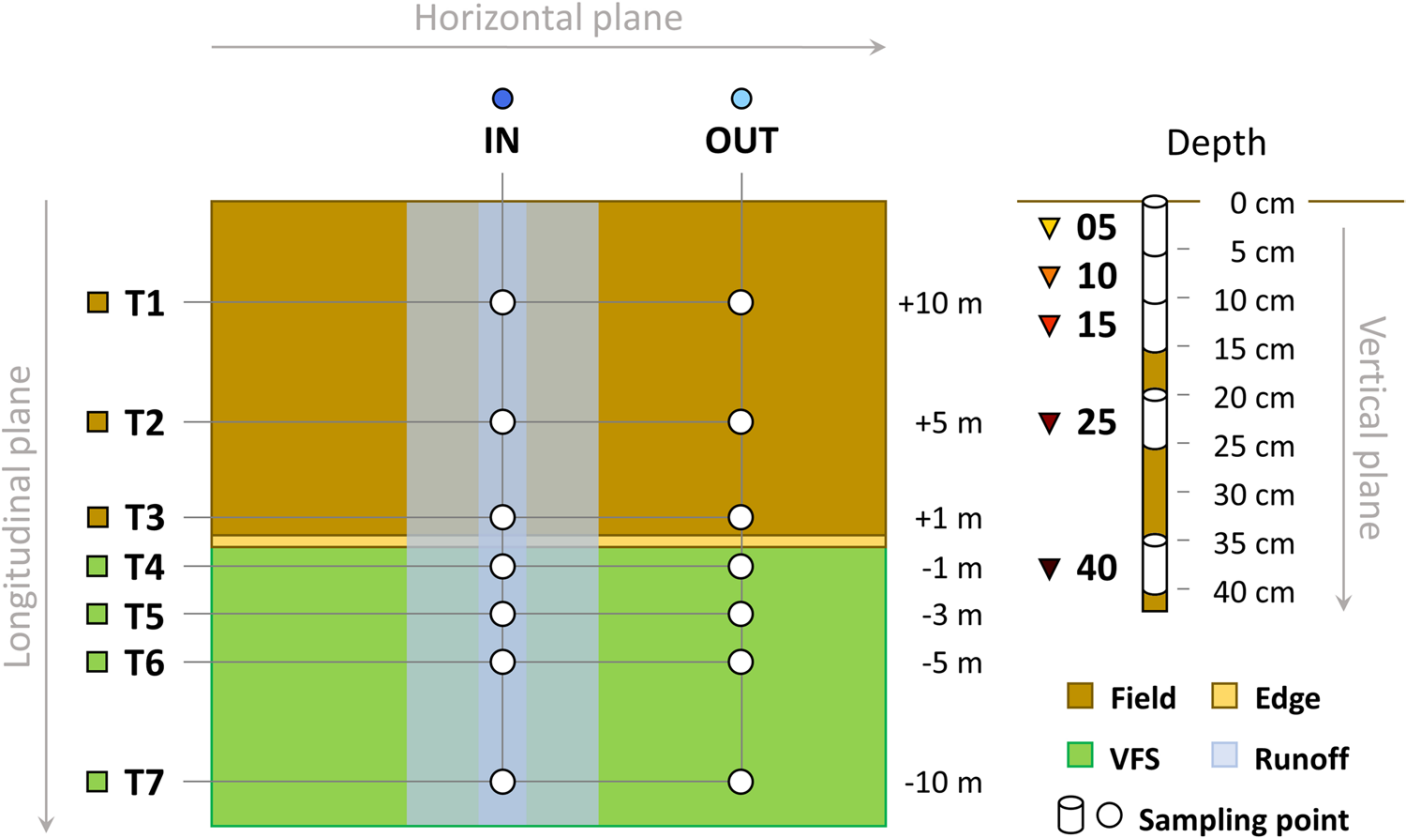
Three-dimensional sampling design comprising two horizontal transects within (*IN*) and outside (*OUT*) of the area of concentrated flow, seven longitudinal transects (*T1*–*T7*) from the field into the VFS, and five depth classes

We sampled six sites from five districts in Lower Austria (Table 1, Fig. 1, Fig. A1-A6, Table A1). The sites were chosen based on a GIS-aided pre-selection of potential locations (i.e., arable fields over grassland with flow accumulation and considerable runoff), in which sub-catchments have been delimitated via a digital elevation model (DEM; 10-m resolution). The final selection was made through an on-site inspection of promising fields after heavy rainfall events. Even though all grassland sites had the functionality of a VFS, some were not intentionally established as designated erosion or runoff mitigation measures. Sampling was adapted after Ramler et al. (2023) and comprised two transects, one in the middle of the area of concentrated runoff and one outside, without a fixed distance (mean distance approx. 10 m). Each transect consisted of seven sampling points (three within the arable field, four within the VFS, spanning over 20 m). Each sampling point consisted of five depth classes (down to 40 cm soil depth), yielding a total of 70 samples per site (Fig. 2). Samples were taken in November 2021, using a custom-built soil core sampler.

**Table 1:**
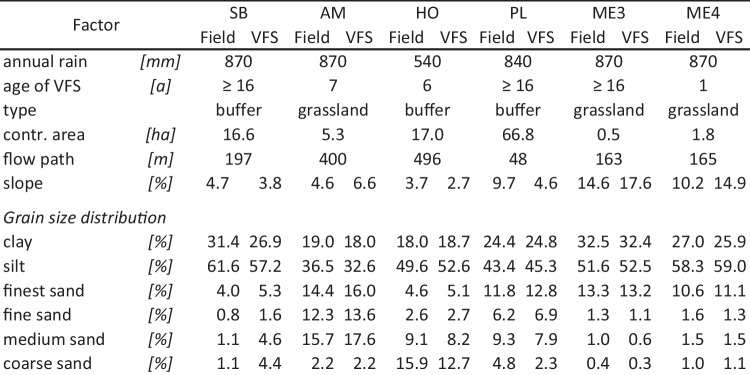
Site characteristics. *Annual rain* was calculated from the monthly sum of precipitation measured by the nearest weather station(s), rounded to the nearest ten. *Age of VFS* indicates the years since the establishment of the VFS. *Type* indicates if the VFS was intentionally set up as a mitigation measure (buffer zone) or if it is regular grassland. *Contr. area* is the catchment area over the VFS sampling grid. Note that this area may also include grassland or forests which have no or a reduced contribution to the flow accumulation (*the majority of the contributing area of PL is grassland). *Flow path* is the approximate length of the longest flow path through the field(s) over the VFS sampling grid. *Slope* indicates the mean slope measured at three locations in the Field and VFS. The *grain size distribution* follows the thresholds (in mm): < 0.002 (clay), 0.063–0.002 (silt), 0.125–0.063 (finest sand), 0.2–0.125 (fine sand), 0.63–0.2 (medium sand), and 2–0.63 (coarse sand)

### Analysis

Before chemical analysis, samples were air-dried and sieved (2 mm). Parameters analyzed include chemical (pH, CaCO_3_, TOC, P, K, Mg, Ca, Fe, Al) and physical parameters (soil texture, dry weight, bulk density). Nutrient fractions were determined using extractants of different strengths (see also Wuenscher et al. 2015): water (e.g., P_H2O_, easily soluble P), calcium-acetate-lactate (e.g., P_CAL_, “plant-available” P), oxalate (e.g., P_ox_, P sorbed to metal oxides), and aqua regia (e.g., P_tot_, total amount of P). The CAL extraction method is routinely used in Austria as a proxy for “plant-available” P. The extraction strength of CAL lies between the Olsen and Mehlich-3 methods, with which CAL is commonly significantly and highly correlated (Zbíral and Němec 2002; Wuenscher et al. 2015). Furthermore, we determined two indices, the degree of P saturation (DPS; van der Zee and van Riemsdijk 1988) and the P sorption index (PSI; Bache and Williams 1971). The DPS estimates how much P is already sorbed to sesquioxides in the soil, a major source of sorption sites for P. It was calculated as:

$$\mathrm{DPS}=\frac{{\mathrm P}_{\mathrm{ox}}}{\propto\times\left({\mathrm{Fe}}_{\mathrm{ox}}\times{\mathrm{Al}}_{\mathrm{ox}}\right)}\times100$$

where *α* is the proportion of oxides that react with P. Following common practice, *α* was set to 0.5 (see Kleinman 2017). The PSI is a proxy for the capacity of soil to absorb P (Bolster et al. 2020). It was calculated as:

$$\mathrm{PSI}=\frac{\mathrm S}{\log\left(\mathrm C\right)}$$

where *S* is the concentration of sorbed P and *C* the equilibrium P concentration in the soil solution. For details on parameter analysis, see Ramler et al. (2023).

For statistical testing, we split the data into four compartments: *Field* and *VFS*, as well as inside (*IN*) and outside (*OUT*) the concentrated flow area. We conducted ANOVAs for each site, followed by Dunn’s post hoc tests to locate statistically significant differences between groups. The statistical significance level *α* was set to 5%. Pearson’s correlation coefficient was calculated to find possible relationships between variables.

Python 3.9.12 embedded in the Spyder 5.2.2 environment was used for statistics and figure generation. Libraries used were *statsmodels*, *scikit_posthoc* (statistical testing), *sklearn* (correlations), *matplotlib*, *seaborn* (plotting), *numpy*, and *pandas* (data handling).

## Results

We have sampled six sites in three dimensions and have analyzed 25 physical and chemical parameters. In the following, we focus on P_H2O_ and P_CAL_ as some of the most important indicators for P (bio-) availability. In addition, P_H2O_ and P_CAL_ are routinely measured for plant fertilization recommendation purposes in Austria. Thus, they are of potential practical use when linked to VFS data. Furthermore, we explore results on DPS and PSI, focusing on trends and differences between IN and OUT, especially in the VFS. Other variables and spatial planes (e.g., depth) are additionally addressed where valuable and appropriate. Results for all variables can be found in the Supplement (Figs. B-G).

**Fig. 3:**
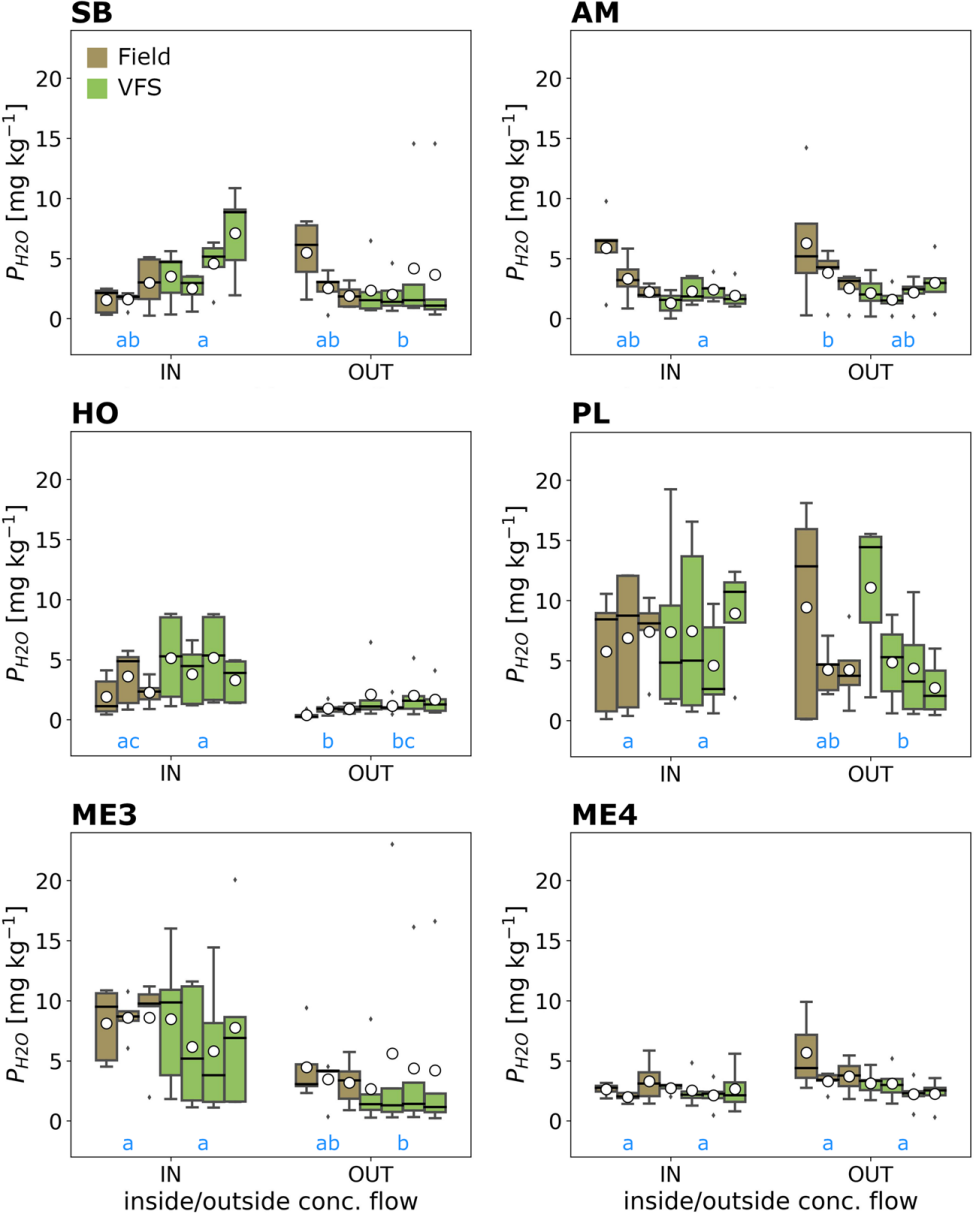
Distribution of water-extractable P (P_H2O_) in field and VFS soils inside (*IN*) and outside (*OUT*) the area of concentrated flow. Boxplots integrate over all depth classes. White circles denote the mean, black lines the median, the box the 25 and 75 percentiles, the whiskers the 5 and 95 percentiles, and the diamonds outliers. The blue letters under the boxplots indicate statistically significant differences between compartments (compartments without significant difference share a letter)

**Fig. 4:**
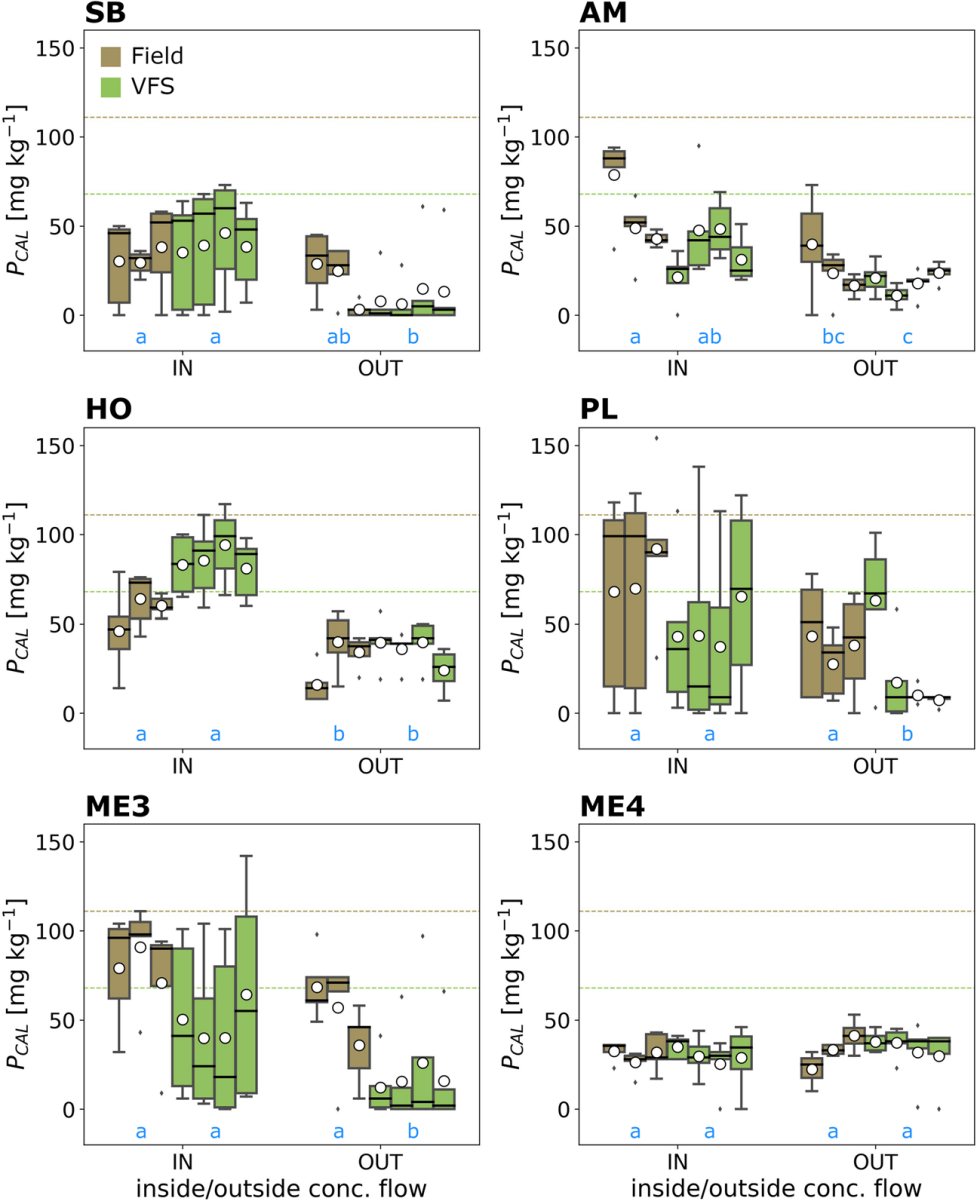
Distribution of calcium-acetate-lactate-extractable P (P_CAL_) in field and VFS soils. The dotted lines indicate the thresholds set by the Austrian Ministry of Agriculture for an adequate P supply in agricultural fields (brown) and grassland (green). See Fig. 3 for further details on symbology

Generally, the concentration and distribution of P_H2O_ and P_CAL_ in the soil, and, thus, absolute and relative values, differed substantially between sites and compartments (Figs. 3 and 4; Table 2). The highest mean concentration for P_H2O_ was found at site ME3 with 8.4 mg kg^−1^ (*Field IN*) and for P_CAL_ at site *HO* with 86 mg kg^−1^ (*VFS IN*), respectively. Site ME4 differed from all others by having almost equal concentrations of P_H2O_ and P_CAL_ throughout all compartments and also showed little variation within a compartment (i.e., between depth classes). As this site constitutes a special case (see “Discussion”), it is excluded in the following and treated separately. All other sites had in common that mean P_CAL_ concentrations were always lower *OUT* compared to *IN* in both the *Field* and *VFS*. This was not entirely true for P_H2O_, being higher *OUT* in the *Field* at site SB and in both the *Field* and *VFS* at site AM. The difference between *VFS IN* and *VFS OUT* was statistically significant at every site for P_CAL_ and for all sites except AM for P_H2O_. The compartment with the lowest P concentrations was commonly *VFS OUT*, except at site HO for P_CAL_ and sites SB and AM for P_H2O_. The highest P concentrations in the *Field* and *VFS* were commonly found *IN*, except for P_H2O_ which had the highest concentration in *Field OUT* at site AM.

**Table 2:**
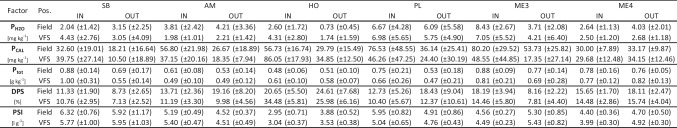
Mean nutrient concentrations and indices values (± standard deviation) in the four compartments. P_H2O_, water-extractable P; P_CAL_, calcium-acetate-lactate-extractable P; P_tot_, aqua regia-extractable P (total P); DPS, degree of phosphorus saturation; PSI, phosphorus sorption index

Mean DPS values ranged from 8.2 to 34.5%. Site HO had by far the highest DPS (in the *Field* and *VFS*) and the highest absolute value at 52.8% (*VFS IN* at the surface layer). The mean DPS was below 20% for all other sites; the lowest mean DPS was found at site SB (*VFS OUT*). Mean PSI ranged from 3.0 to 6.3 (l g^−1^, however, the unit of the PSI, is essentially meaningless). Only site HO had mean PSI values lower than 4.

**Fig. 5:**
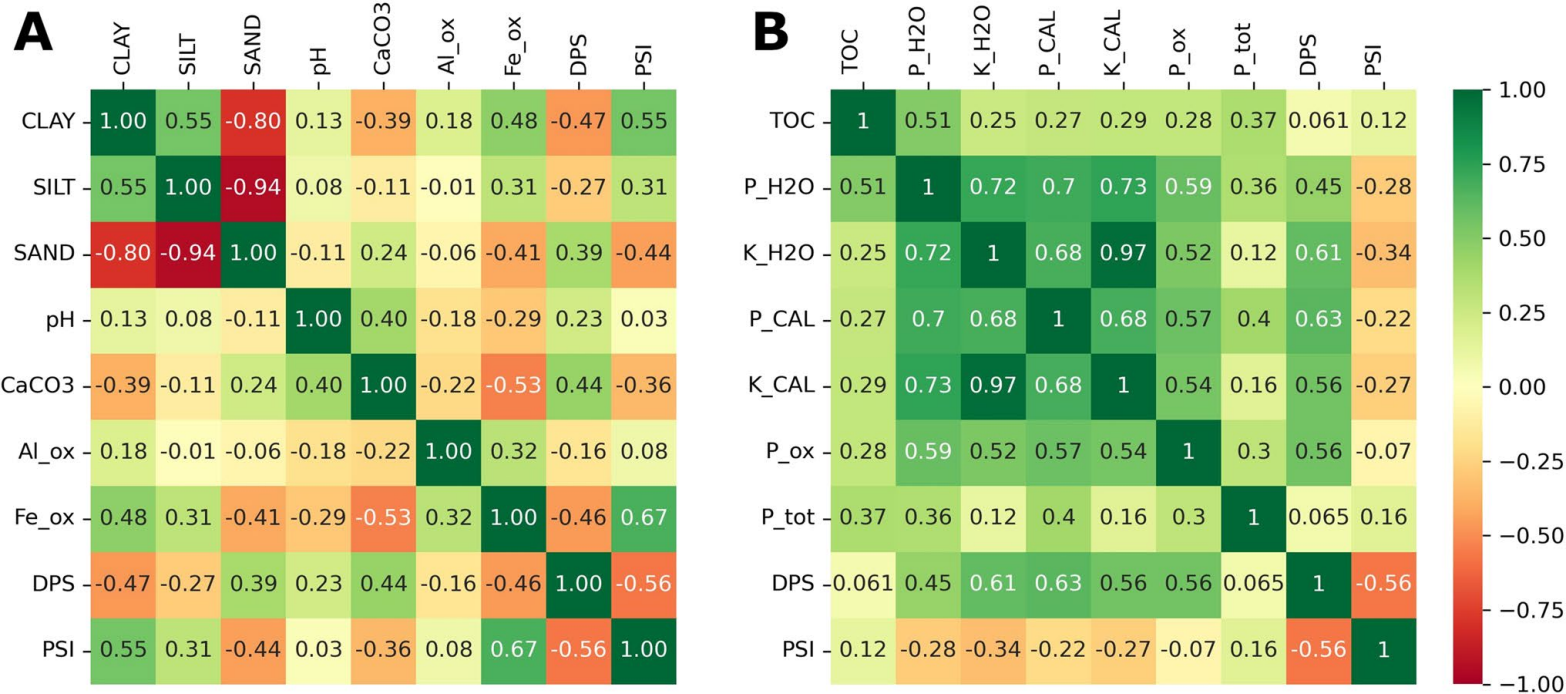
Heatmap of Pearson’s correlation coefficients (*r*) for selected physical and chemical parameters (**A**) and nutrients and indices (**B**). ox, oxalate-extractable elements; H_2_O, water-extractable elements; CAL, calcium-acetate-lactate-extractable elements; DPS, degree of phosphorus saturation; PSI, phosphorus sorption index

Overall trends between compartments and sites were comparable for potassium (K) and other P pools (Fig. B2-G2). Nutrient variables and indices were all significantly and positively correlated, with the exception of P_tot_ and DPS (Fig. 5). Pearson’s *r* was higher than 0.5 for all P and K variable combinations, except for P_tot_ which had its highest correlation of 0.4 with P_CAL_. DPS was also well correlated with P and K variables, except for P_tot_. PSI, in turn, was largely uncorrelated with chemical elements, but had a positive Pearson’s *r* with clay content of 0.55 and Fe_ox_ concentration of 0.67. DPS and PSI were negatively correlated with − 0.56.

## Discussion

### Site-specificity of nutrient distribution in the soil

A theoretical baseline situation for a field-VFS system under sheet flow and with a well-established, longstanding vegetation would consist of a probably rather high amount of nutrients (e.g., P_CAL_) in the field due to fertilization and, vice versa, a lower amount in the VFS, which receives field runoff but no direct fertilization. However, the nutrient distribution of the same system under concentrated flow conditions would change drastically. In the VFS, outside the concentrated flow area (*OUT*), we would expect much lower nutrient levels, as this part would receive neither direct nor indirect fertilization. Nutrient concentrations in the field would be similar to baseline values or decrease slightly due to a washing-out effect. Inside the concentrated flow area (*IN*), nutrient levels in the field would be lower compared with both the baseline situation and the field area outside the concentrated flow, due to erosion and a further pronounced washing out. However, if there is a backlog of water at the field-VFS transition, and, thus, sediment deposition and infiltration, nutrient concentrations could increase (locally) as well. In the VFS, we would expect a substantial increase in nutrient levels due to the nutrient-enriched runoff it receives and the inability of this small remaining effective area of the VFS to process all incoming nutrients. Furthermore, the nutrient distribution in the soil would likely show longitudinal (e.g., higher nutrient levels closer to the field-VFS transition) as well as vertical gradients (e.g., decrease of nutrient levels with depth).

We may identify these theoretical patterns in our data, although substantial variation exists between sites and compartments (*Field*, *VFS*, *IN*, *OUT*). This highlights the site-specificity of the processes that ultimately determine VFS retention success. These are linked to a multitude of abiotic and biotic factors, such as tillage, cropping, fertilization, climate, soil texture, or vegetation type (Prosser et al. 2020; Stutter et al. 2021; Ramler et al. 2023). For the purpose of this study, P_CAL_ is arguably the most important parameter, as this nutrient fraction is still relatively easily soluble (and thus “bio-” or “plant-available”), but not as volatile as P_H2O_. Furthermore, it is the basis for fertilizer recommendations by the Austrian Ministry of Agriculture (Baumgarten 2022). All but one site showed substantially higher P_CAL_ concentration inside the area of concentrated flow compared to outside — both in the field and in the VFS. Three sites showed very low nutrient levels in the *VFS OUT* compartment, as expected, while the other three sites (AM, HO, ME4) had elevated nutrient contents, similar to the *Field OUT* compartment. This is primarily caused by the age of the buffer strip (i.e., the time since establishment), most apparent for site ME4, at which the VFS was established just the year before sampling. Before, the area of the VFS was also used as arable land, managed identically to the adjacent field. Consequently, the nutrient distribution in the soil is still very homogenous, due to tillage, and nutrient levels in the VFS are similar to the field due to long-standing fertilization. At sites AM and HO, a VFS was established for 7 and 6 years, respectively, long enough for differences between *IN* and *OUT* to develop. It can be presumed that, as time progresses, P_CAL_ values outside the concentrated flow in the VFS will approximate the other sites, which exhibit baseline values for unfertilised grassland (e.g., Bohner 2005). Similar results were obtained for P_H2O_ and nearly all other nutrients and nutrient pools (Fig. 3, Fig. B2-G2).

Within the VFSs, P_CAL_ concentrations *IN* were, on average, 105% higher than *OUT* (up to 279% at site *SB*). This shows that VFS can accumulate substantial amounts of P, even if they do not receive direct fertilization. Elevated nutrient levels *OUT* (e.g., at sites ME3 and SB) were restricted to the uppermost layer, with a prominent difference to the following sampling depths. Nutrient accumulation inside the concentrated flow area also occurred in subsurface layers, at site HO even down to the deepest sampling layer (40 cm). Infiltration of runoff water and transport to deeper soil layers is, in principle, a desirable process, as it increases the effective soil volume that can contribute to nutrient retention, processing, and cycling (Ramler et al. 2022). At site HO, infiltration was fostered by a high sand content at the surface. Nevertheless, high concentrations in the deep also increase the risk of leaching nutrients into aquifers and groundwater (Djodjic et al. 2004; Andersson et al. 2015). Promoting deep-rooting plant species in the VFS could enhance infiltration and nutrient uptake from subsurface soil layers (Delorme et al. 2000; Sheng et al. 2021). If the buffer vegetation is sub-optimal (e.g., plant species/vegetation type with insufficient nutrient uptake), not managed properly (e.g., not regularly cut and removed), or the VFS design itself flawed (e.g., amount of nutrient input not matching buffer shape and extent), nutrients will accumulate, increasing the risk of P export. The higher mean P values (all P fractions) at *VFS IN* compared to *Field IN* at sites SB and HO already point in this direction.

Concentrations of P_H2O_ and P_CAL_ also tended to be higher *IN* than *OUT* in the field (Fig. 4), especially at surface layers. Higher nutrient contents close to the field edge were also reported by other studies. They were commonly attributed to a barrier effect of the vegetation or the formation of berms, ridges, and similar structures that cause a backlog of water, promoting sediment deposition and infiltration (Stutter et al. 2009; Habibiandehkordi et al. 2019). This could have played a role in our study, although sites PL, ME3, and ME4 were probably too steep for this to happen. Higher nutrient levels may also be influenced by the fertilization procedure on the field. For instance, at sites PL and ME3, the *IN* sampling transect was close to the lateral field edge. It is possible that this area received more fertilizer than more inward areas, for instance due to a slower initial tractor speed. Analogously, field areas may receive more nutrients if the field shape necessitates a slower operational speed or making turns, leading to a fertilizer spread overlap (Kharel et al. 2020a, b). The wedge-like area at site HO would probably require such actions. Inhomogenous nutrient distribution in the field soil may also arise due to imprecise applicators (e.g., bulk spreaders) or other stochastic effects (Mallarino 1996; Gyldengren et al. 2020). The nutrient export risk increases disproportionally if such areas overlap with concentrated flow pathways. The adaption of precision farming techniques can help mitigate this issue, affecting not only nutrient export but also fertilizer costs and yields (Søgaard and Kierkegaard 1994; Pedersen et al. 2020).

The Austrian Ministry of Agriculture recommends P levels (P_CAL_) within the range of 47–111 mg kg^−1^ for an adequate P supply of field soils (Baumgarten 2022). Above this threshold, phosphorus concentrations would be considered (too) high, and fertilization should cease. All sites were within this range, except for site PL, which exceeded the limit at surface layers (0–10 cm) inside the concentrated flow area. All sites exceeded the recommended P range for grassland (47–68 mg kg^−1^) at one or more sampling points. At site HO, all sampling points had higher mean P_CAL_ concentrations than recommended for grassland and some surface samples even exceeded the recommendations for cropland. Despite the partially high P contents in VFS soils, mean DPS values were low, ranging between 5 and 20%. An exception was, again, site HO, with a mean DPS of 34.5% at VFS IN and a maximum of 52.8%. All sampling points were above 25% DPS at site HO, commonly considered an environmental threshold value above which the P leaching risk increases substantially (Bajouco et al. 2020; Elbasiouny et al. 2020). Despite comparably high P contents, these high DPS values that are not met by any other sampling site are linked to the P sorption capacity and soil composition. Metal oxides, clay minerals, and humic substances are all important sorption sites for P (Holford 1997; Gérard 2016; Weihrauch and Opp 2018). Site HO had the lowest concentrations of Fe_ox_ and Al_ox_ and a low proportion of clay and TOC content — variables that can be considered proxies for these sorption sites (Börling et al. 2001). Consequently, site HO has a low capacity for P uptake, which is also reflected in the PSI, which was the lowest of all sites. High P content and low P sorption capacity inevitably lead to a high P saturation. Soil composition should, thus, be considered more strongly during VFS design. Soils akin to site HO have a diminished retention capacity, which implies that they need to be managed more closely (e.g., more frequent cuts, plant species with high nutrient uptake), or that more emphasis should be given to in-field mitigation measures that prevent erosion in the first place (e.g., contour cropping, less erosion prone crops).

### Correlation of soil variables

All P (and K) variables were positively correlated, meaning that an increase or decrease of one P fraction is at least partly mirrored by the others. Their correlation with total P (P_tot_) was, however, only moderate to very weak (Pearson’s *r* < 0.4). Generally, P occurs in a continuum in the soil along a (bio-)availability gradient, i.e., P that is more or less strongly bound to other substances (Weihrauch and Opp 2018). Although no reagents exist to determine all P, strong chemicals like aqua regia can extract most of the sediment bound P. As such, it is less affected by soil–water interactions within the runoff than the other more easily soluble P fractions. Similar findings of moderate to good correlations of different soil test P (STP) fractions with each other, but only weak to no correlation with total P, were also found by others (Zbíral and Němec 2002; Quinton et al. 2003; Wuenscher et al. 2015), though there are also studies with higher correlations between STP and total P (Pautler and Sims 2000). The overall trends of P correlations follow Wuenscher et al. (2015), who also analyzed Austrian soils. However, P_CAL_ and P_ox_ were substantially higher correlated with P_H2O_ and much weaker correlated with total P in our study. At any rate, the high variation found between studies implies that the potential to derive one or more P fractions from measuring another is limited and site-specific.

DPS and PSI showed a moderate negative correlation. Only a few studies have analyzed the relationship between DPS and PSI. Theoretically, the two indices are independent of another; nevertheless, the likelihood of a soil with a low capacity for P uptake to be saturated is higher by default. Interestingly, Messiga et al. (2021) found a high positive correlation (Pearson’s *r* = 0.88) in a study of Canadian soils. However, they used a different method for PSI determination, by calculating the quotient of Mehlich-3-extractable P and Al (Khiari et al. 2000). As the DPS is also derived from P and Al measurements, it is not surprising that this method leads to a correlation of DPS and PSI. In the literature, there are several suggested approaches how to calculate PSI and DPS (e.g., Wang et al. 2016; Blombäck et al. 2021). This is caused by soil-specific constraints (e.g., alkaline vs. acidic soils), but also by a desire for a quick and inexpensive determination, preferably using affordable and already established laboratory methods. Their accuracy and validity should, however, be examined. The soils used by Messiga et al. (2021) had a high legacy P content (mean TP = 1.9 g kg^−1^) and differed from our samples also by a much lower pH value (ranging from 5.1 to 5.9), which affects the presence of metal hydroxides and available sorption sites (Nyamaizi et al. 2022). The relationship between PSI and DPS is, thus, probably also site-specific to some extent.

Calcium carbonate (CaCO_3_) was positively correlated with DPS and pH. This is in accordance with Weng et al. (2012), who found that pH, Ca concentration, and organic matter were the most important factors controlling P sorption to iron oxides. Higher Ca concentrations imply a higher amount of P absorbed, leading to a higher DPS. In turn, CaCO_3_ concentration was negatively correlated with Al and Fe oxides and, thus, PSI.

## Conclusions

Our hypothesis that concentrated flow would lead to higher nutrients inside the area of concentrated flow was met. At two sites, levels of P_CAL_ were even higher in the VFS than in the field. This shows that field runoff, especially in the case of concentrated flow, can lead to nutrient accumulation — and, thus, an increased export risk — in a VFS, even if it does not receive any direct fertilization. Consequently, VFSs need to be monitored to prevent a pronounced nutrient build-up in the first place or to take countermeasures. Furthermore, the nutrient distribution in the field and VFS soils was highly site-specific. The capacity of a VFS to effectively retain and process incoming sediment and nutrients is influenced by a multitude of factors related to the VFS itself (e.g., soil texture, physico-chemical composition, vegetation, age), climate (e.g., frequency and intensity of rainfall), and agricultural practice (e.g., cropping, fertilization). Accordingly, the planning and implementation of VFS must become more sophisticated, with a priori measurements and bespoke designs capable of processing the actual sediment and nutrient load.

### Supplementary Information

Below is the link to the electronic supplementary material.

Supplementary file1 (PDF 10358 KB)

## Data Availability

The raw data are available from the authors upon reasonable request.

## References

[CR1] Andersson H, Bergström L, Ulén B et al (2015) The role of subsoil as a source or sink for phosphorus leaching. J Environ Qual 44:535–544. 10.2134/jeq2014.04.0186 26023972 10.2134/jeq2014.04.0186

[CR2] Bache BW, Williams EG (1971) A phosphate sorption index for soils. J Soil Sci 22:289–301. 10.1111/j.1365-2389.1971.tb01617.x 10.1111/j.1365-2389.1971.tb01617.x

[CR3] Bajouco R, Pinheiro J, Pereira B et al (2020) Risk of phosphorus losses from Andosols under fertilized pasture. Environ Sci Pollut Res 27:19592–19602. 10.1007/s11356-020-08492-y 10.1007/s11356-020-08492-y 32219657

[CR4] Baumgarten A (ed) (2022) Richtlinie für die sachgerechte Düngung im Ackerbau und Grünland. Bundesministerium für Landwirtschaft, Regionen und Tourismus, Vienna, Austria

[CR5] Blombäck K, Bolster CH, Lindsjö A et al (2021) Comparing measures for determination of phosphorus saturation as a method to estimate dissolved P in soil solution. Geoderma 383:114708. 10.1016/j.geoderma.2020.114708 10.1016/j.geoderma.2020.114708

[CR6] Bohner A (2005) Soil chemical properties as indicators of plant species richness in grassland communities. In: Lillak R, Viiralt R, Linke A, Geherman V (eds) Integrating efficient grassland farming and biodiversity. EGF, Tartu, Estonia, pp 65–68

[CR7] Bolster CH, McGrath JM, Rosso E, Blombäck K (2020) Evaluating the effectiveness of the phosphorus sorption index for estimating maximum phosphorus sorption capacity. Soil Sci Soc Am J 84:994–1005. 10.1002/saj2.20078 10.1002/saj2.20078

[CR8] Börling K, Otabbong E, Barberis E (2001) Phosphorus sorption in relation to soil properties in some cultivated swedish soils. Nutr Cycl Agroecosystems 59:39–46. 10.1023/A:1009888707349 10.1023/A:1009888707349

[CR9] Correll DL (1998) The role of phosphorus in the eutrophication of receiving waters: a review. J Environ Qual 27:261–266. 10.2134/jeq1998.00472425002700020004x 10.2134/jeq1998.00472425002700020004x

[CR10] Delorme TA, Angle JS, Coale FJ, Chaney RL (2000) Phytoremediation of phosphorus-enriched soils. Int J Phytoremediation 2:173–181. 10.1080/15226510008500038 10.1080/15226510008500038

[CR11] Djodjic F, Börling K, Bergström L (2004) Phosphorus leaching in relation to soil type and soil phosphorus content. J Environ Qual 33:678–684. 10.2134/jeq2004.6780 15074820 10.2134/jeq2004.6780

[CR12] Dosskey MG, Helmers MJ, Eisenhauer DE et al (2002) Assessment of concentrated flow through riparian buffers. J Soil Water Conserv 57:336 LP – 343

[CR13] Elbasiouny H, Elbehiry F, El-Ramady H, Brevik EC (2020) Phosphorus availability and potential environmental risk assessment in alkaline soils. Agriculture 10:172. 10.3390/agriculture10050172

[CR14] Gérard F (2016) Clay minerals, iron/aluminum oxides, and their contribution to phosphate sorption in soils — a myth revisited. Geoderma 262:213–226. 10.1016/j.geoderma.2015.08.036 10.1016/j.geoderma.2015.08.036

[CR15] Gyldengren J, Greve MB, Skou-Nielsen N et al (2020) Field scale agronomic and environmental consequences of overlapping N fertilizer application by disc spreaders. F Crop Res 255:107901. 10.1016/j.fcr.2020.107901 10.1016/j.fcr.2020.107901

[CR16] Habibiandehkordi R, Lobb DA, Owens PN, Flaten DN (2019) Effectiveness of vegetated buffer strips in controlling legacy phosphorus exports from agricultural land. J Environ Qual 48:314–321. 10.2134/jeq2018.04.0129 30951107 10.2134/jeq2018.04.0129

[CR17] Hancock G, Hamilton SE, Stone M et al (2015) A geospatial methodology to identify locations of concentrated runoff from agricultural fields. JAWRA J Am Water Resour Assoc 51:1613–1625. 10.1111/1752-1688.12345 10.1111/1752-1688.12345

[CR18] Helmers MJ, Eisenhauer DE, Dosskey MG et al (2005) Flow pathways and sediment trapping in a field-scale vegetative filter. Trans ASAE 48:955–968. 10.13031/2013.18508 10.13031/2013.18508

[CR19] Holford ICR (1997) Soil phosphorus: its measurement, and its uptake by plants. Soil Res 35:227–240 10.1071/S96047

[CR20] Jarvie HP, Sharpley AN, Spears B et al (2013) Water quality remediation faces unprecedented challenges from “legacy phosphorus.” Environ Sci Technol 47:8997–8998. 10.1021/es403160a 23931665 10.1021/es403160a

[CR21] Kharel TP, Ashworth AJ, Shew A et al (2020a) Tractor guidance improves production efficiency by reducing overlaps and gaps. Agric Environ Lett 5:e20012. 10.1002/ael2.20012 10.1002/ael2.20012

[CR22] Kharel TP, Owens PR, Ashworth AJ (2020b) Tractor path overlap is influenced by field shape and terrain attributes. Agric Environ Lett 5:e20027. 10.1002/ael2.20027 10.1002/ael2.20027

[CR23] Khiari L, Parent LE, Pellerin A et al (2000) An agri-environmental phosphorus saturation index for acid coarse-textured soils. J Environ Qual 29:1561–1567. 10.2134/jeq2000.00472425002900050024x 10.2134/jeq2000.00472425002900050024x

[CR24] Kleinman PJA (2017) The persistent environmental relevance of soil phosphorus sorption saturation. Curr Pollut Reports 3:141–150. 10.1007/s40726-017-0058-4 10.1007/s40726-017-0058-4

[CR25] Mallarino AP (1996) Spatial variability patterns of phosphorus and potassium in no-tilled soils for two sampling scales. Soil Sci Soc Am J 60:1473–1481. 10.2136/sssaj1996.03615995006000050027x 10.2136/sssaj1996.03615995006000050027x

[CR26] Messiga AJ, Lam C, Li Y (2021) Phosphorus saturation index and water-extractable phosphorus in high-legacy phosphorus soils in southern British Columbia, Canada. Can J Soil Sci 101:365–377. 10.1139/cjss-2020-0129 10.1139/cjss-2020-0129

[CR27] Nyamaizi S, Messiga AJ, Cornelis J-T, Smukler SM (2022) Effects of increasing soil pH to near-neutral using lime on phosphorus saturation index and water-extractable phosphorus. Can J Soil Sci 102:929–945. 10.1139/cjss-2021-0197 10.1139/cjss-2021-0197

[CR28] Pankau RC, Schoonover JE, Williard KWJ, Edwards PJ (2012) Concentrated flow paths in riparian buffer zones of southern Illinois. Agrofor Syst 84:191–205. 10.1007/s10457-011-9457-5 10.1007/s10457-011-9457-5

[CR29] Pautler MC, Sims JT (2000) Relationships between soil test phosphorus, soluble phosphorus, and phosphorus saturation in delaware soils. Soil Sci Soc Am J 64:765–773. 10.2136/sssaj2000.642765x 10.2136/sssaj2000.642765x

[CR30] Pedersen SM, Pedersen MF, Ørum JE et al (2020) Economic, environmental and social impacts. In: Buttafuoco G, Khosla R et al (eds) Castrignanò A. Things and decision support for precision smart farming. Academic Press, Agricultural Internet of, pp 279–330

[CR31] Prosser RS, Hoekstra PF, Gene S et al (2020) A review of the effectiveness of vegetated buffers to mitigate pesticide and nutrient transport into surface waters from agricultural areas. J Environ Manage 261:110210. 10.1016/j.jenvman.2020.110210 32148280 10.1016/j.jenvman.2020.110210

[CR32] Quinton JN, Strauss P, Miller N et al (2003) The potential for soil phosphorus tests to predict phosphorus losses in overland flow. J Plant Nutr Soil Sci 166:432–437. 10.1002/jpln.200321122 10.1002/jpln.200321122

[CR33] Ramler D, Stutter M, Weigelhofer G et al (2022) Keeping up with phosphorus dynamics: overdue conceptual changes in vegetative filter strip research and management. Front Environ Sci 10:1–8. 10.3389/fenvs.2022.764333 10.3389/fenvs.2022.764333

[CR34] Ramler D, Inselsbacher E, Strauss P (2023) A three-dimensional perspective of phosphorus retention across a field-buffer strip transition. Environ Res 233:116434. 10.1016/j.envres.2023.116434 37343753 10.1016/j.envres.2023.116434

[CR35] Schindler DW, Carpenter SR, Chapra SC et al (2016) Reducing phosphorus to curb lake eutrophication is a success. Environ Sci Technol 50:8923–8929. 10.1021/acs.est.6b02204 27494041 10.1021/acs.est.6b02204

[CR36] Sharpley AN, Jarvie HP, Buda A et al (2013) Phosphorus legacy: overcoming the effects of past management practices to mitigate future water quality impairment. J Environ Qual 42:1308–1326. 10.2134/jeq2013.03.0098 24216410 10.2134/jeq2013.03.0098

[CR37] Sheng L, Zhang Z, Xia J et al (2021) Impact of grass traits on the transport path and retention efficiency of nitrate nitrogen in vegetation filter strips. Agric Water Manag 253:106931. 10.1016/j.agwat.2021.106931 10.1016/j.agwat.2021.106931

[CR38] Shrestha P, Williard KWJ, Schoonover JE, Park L (2018) Prevalence of concentrated flow paths in agricultural fields in southern Illinois. Water, Air, Soil Pollut 229:198. 10.1007/s11270-018-3841-y 10.1007/s11270-018-3841-y

[CR39] Søgaard HT, Kierkegaard P (1994) Yield reduction resulting from uneven fertilizer distribution. Trans ASAE 37:1749–1752. 10.13031/2013.28262 10.13031/2013.28262

[CR40] Stutter MI, Langan SJ, Lumsdon DG (2009) Vegetated buffer strips can lead to increased release of phosphorus to waters: a biogeochemical assessment of the mechanisms. Environ Sci Technol 43:1858–1863. 10.1021/es8030193 19368183 10.1021/es8030193

[CR41] Stutter MI, Chardon WJ, Kronvang B (2012) Riparian buffer strips as a multifunctional management tool in agricultural landscapes: introduction. J Environ Qual 41:297–303. 10.2134/jeq2011.0439 22370391 10.2134/jeq2011.0439

[CR42] Stutter M, Costa FB, Ó hUallacháin D (2021) The interactions of site-specific factors on riparian buffer effectiveness across multiple pollutants: a review. Sci Total Environ 798:149238. 10.1016/j.scitotenv.2021.149238 10.1016/j.scitotenv.2021.149238 34325145

[CR43] Ulén B, Bechmann M, Fölster J et al (2007) Agriculture as a phosphorus source for eutrophication in the north-west European countries, Norway, Sweden, United Kingdom and Ireland: a review. Soil Use Manag 23:5–15. 10.1111/j.1475-2743.2007.00115.x 10.1111/j.1475-2743.2007.00115.x

[CR44] van der Zee SEATM, van Riemsdijk WH (1988) Model for long-term phosphate reaction kinetics in soil. J Environ Qual 17:35–41. 10.2134/jeq1988.00472425001700010005x 10.2134/jeq1988.00472425001700010005x

[CR45] Wang YT, Zhang TQ, O’Halloran IP et al (2016) A phosphorus sorption index and its use to estimate leaching of dissolved phosphorus from agricultural soils in Ontario. Geoderma 274:79–87. 10.1016/j.geoderma.2016.04.002 10.1016/j.geoderma.2016.04.002

[CR46] Weihrauch C, Opp C (2018) Ecologically relevant phosphorus pools in soils and their dynamics: the story so far. Geoderma 325:183–194. 10.1016/j.geoderma.2018.02.047 10.1016/j.geoderma.2018.02.047

[CR47] Weng L, Van Riemsdijk WH, Hiemstra T (2012) Factors controlling phosphate interaction with iron oxides. J Environ Qual 41:628–635. 10.2134/jeq2011.0250 22565244 10.2134/jeq2011.0250

[CR48] Wuenscher R, Unterfrauner H, Peticzka R, Zehetner F (2015) A comparison of 14 soil phosphorus extraction methods applied to 50 agricultural soils from Central Europe. Plant, Soil Environ 61:86–96. 10.17221/932/2014-PSE 10.17221/932/2014-PSE

[CR49] Zbíral J, Němec P (2002) Comparison of Mehlich 2, Mehlich 3, CAL, Egner, Olsen, and 0.01 M CaCl2 extractants for determination of phosphorus in soils. Commun Soil Sci Plant Anal 33:3405–3417. 10.1081/CSS-120014534 10.1081/CSS-120014534

