# Supplementary material for: Site matters: site-specific factors control phosphorus retention in buffer strip soils under concentrated field runoff
Source: Environ Sci Pollut Res Int. 2024 Jul 17;31(35):48154–63. doi: 10.1007/s11356-024-34383-7 (PMC11297899; doi:10.1007/s11356-024-34383-7)
Supplement: Supplementary file 1 — Supplementary file1 (PDF 10358 KB) [file 11356_2024_34383_MOESM1_ESM.pdf]

Site *SB*

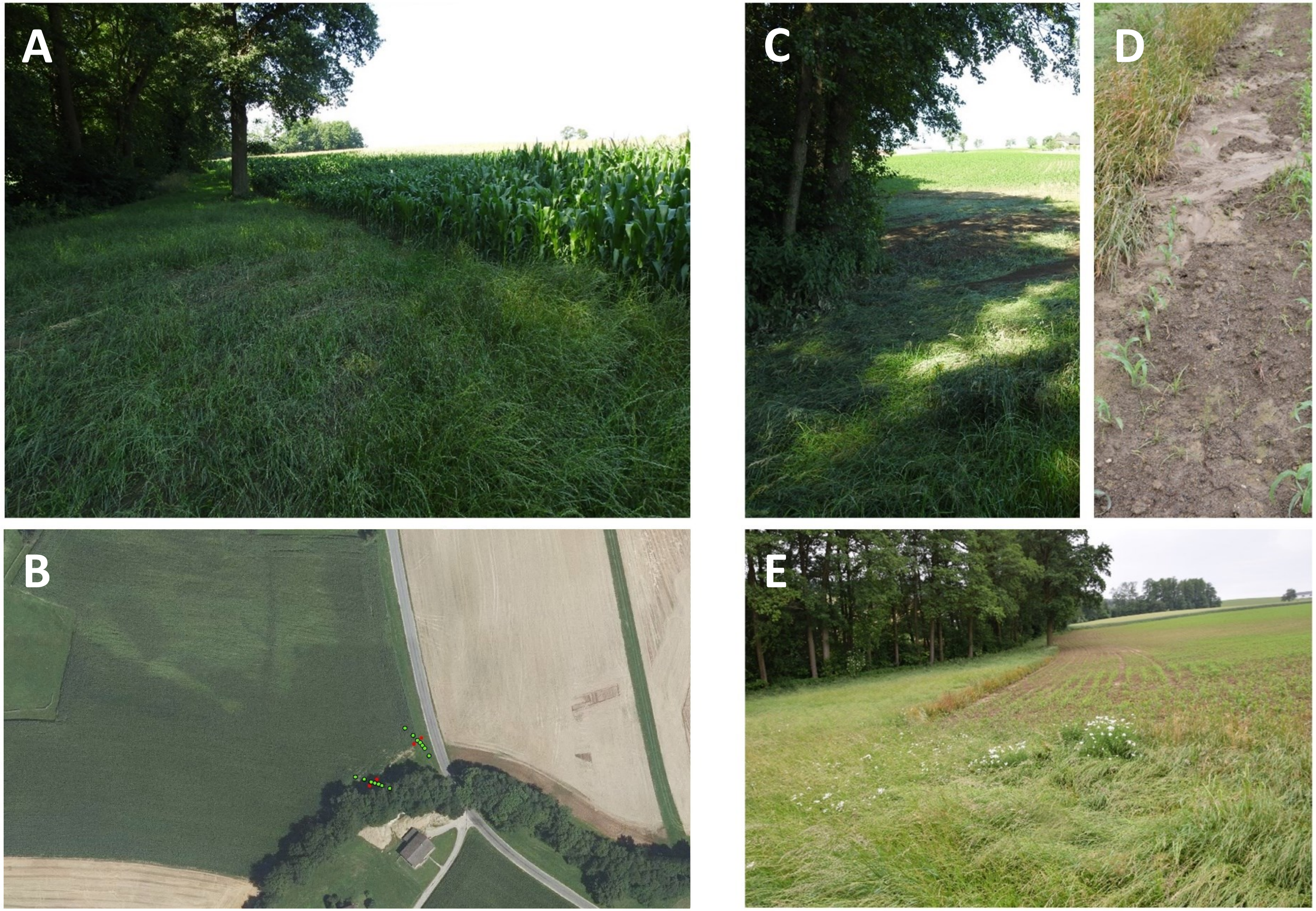


**Fig. A1** Site SB. [A] Situation after a heavy rainfall event (July 2021). [B] Orthophoto of the VFS and the contributing agricultural area (2020). Green dots indicate the sampling poi nts; red dots the actual field/VFS border. [C+D] Detailed view of deposited sediment in the VFS and at the field/VFS transistion (July 2021). [E] overview of site SB (June 2020).

Site AM

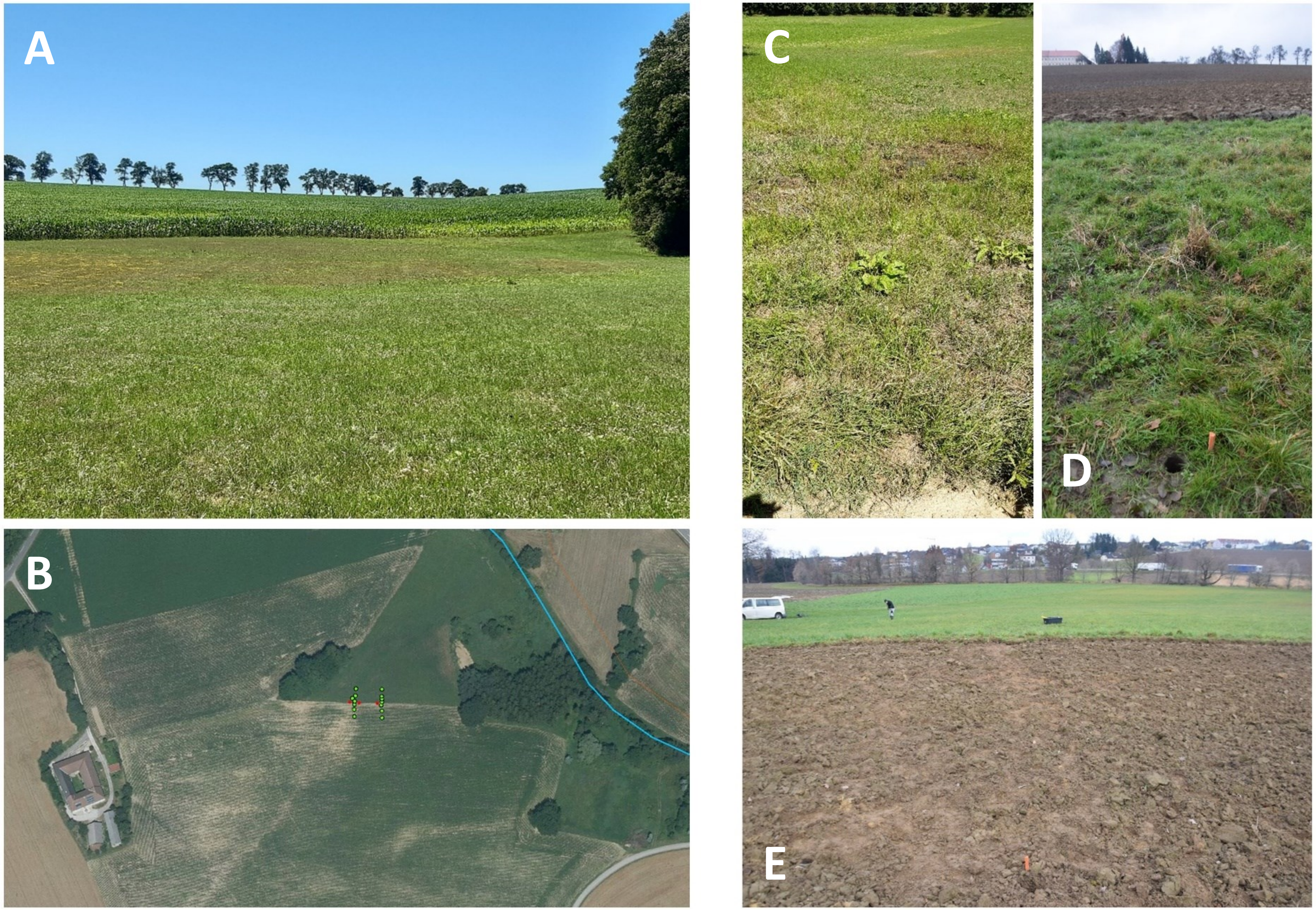


**Fig. A2** Site AM. [A] Situation after a heavy rainfall event (July 2021). [B] Orthophoto of the VFS and the contributing agricultural area (2019). Green dots indicate the sampling points; red dots the actual field/VFS border. [C+D] Detailed view of deposited sediment in the VFS (July 2021). [E] View from the field into the VFS during sampling (November 2021).

Site *HO*

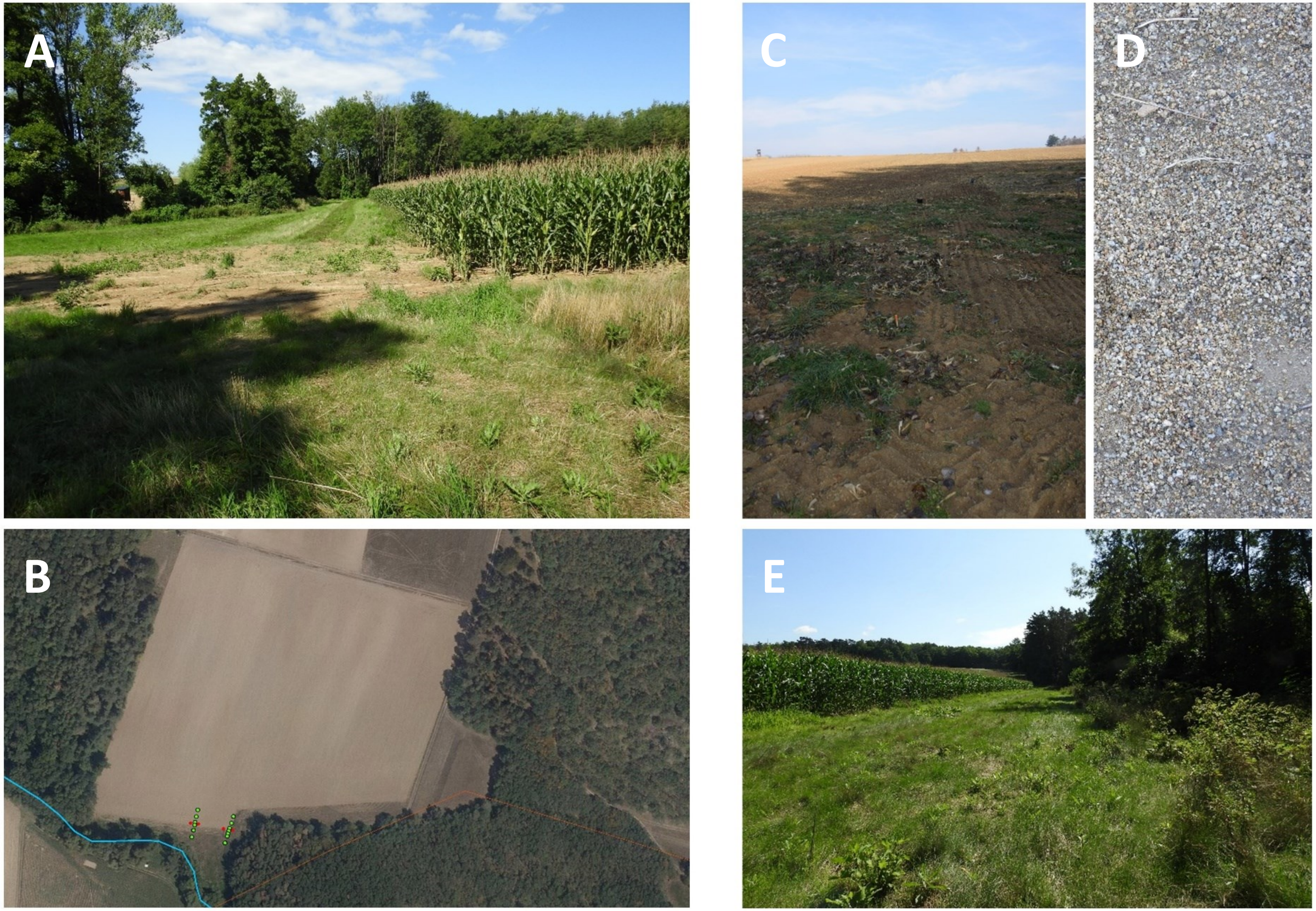


**Fig. A3** Site HO. [A] Situation after a heavy rainfall event (August 2021). [B] Orthophoto of the VFS and the contributing agricultural area (2018). Green dots indicate the sampling points; red dots the actual field/VFS border. [C] Detailed view of deposited sediment in the VFS (November 2021). [D] Close-up of the deposited sediment and its sandy composition. [E] Overview of site HO (August 2021).

Site *PL*

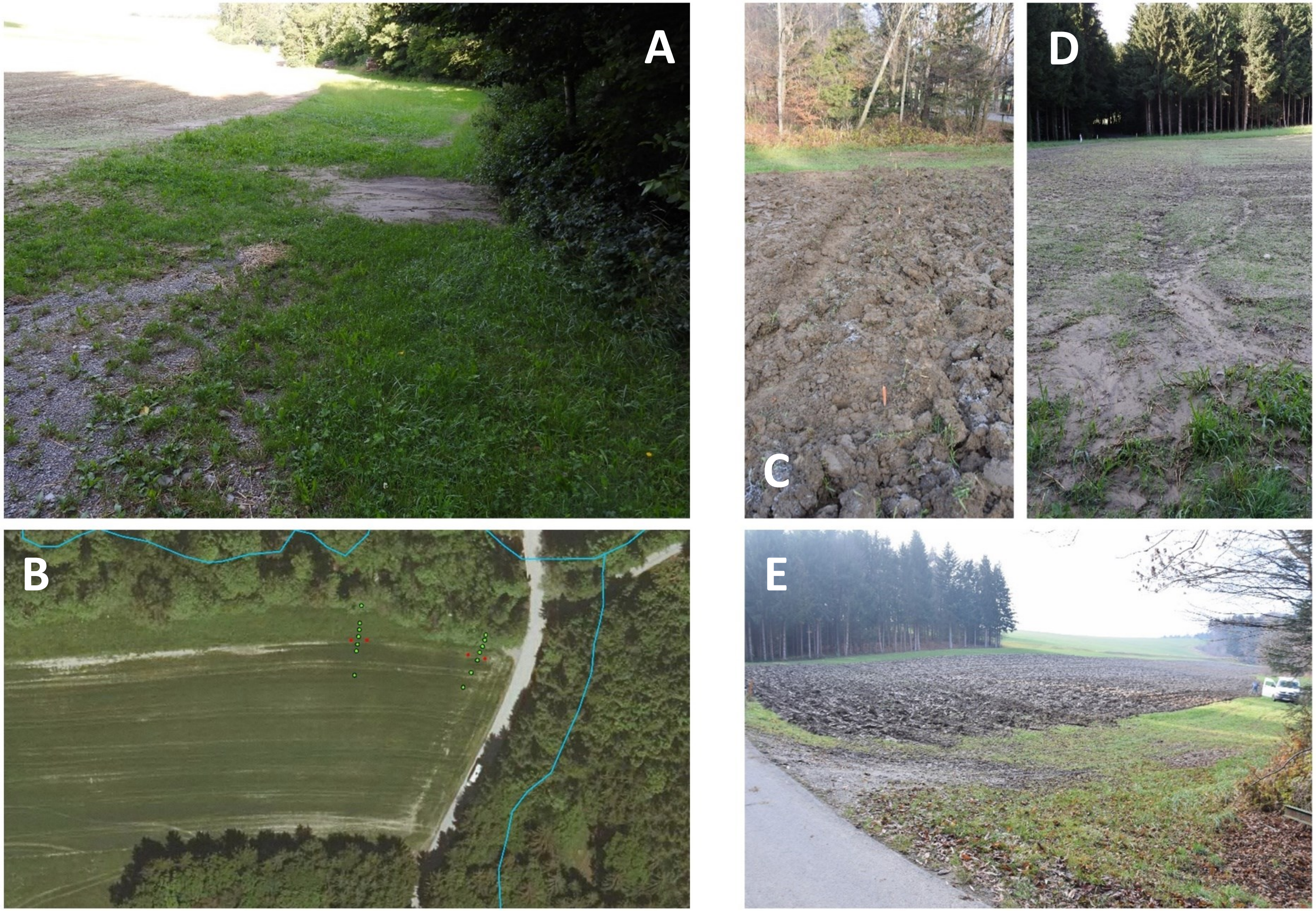


**Fig. A4** Site PL. [A] Situation after a heavy rainfall event (July 2021). [B] Orthophoto of the VFS and the contributing agricultural area (2017). Green dots indicate the sampling points; red dots the actual field/VFS border. [C] View from the field into the VFS during sampling (November 2021). [D] Detailed view of the erosion pathway and deposited sediment. [E] Overview of site PL (November 2021).

Site *ME3*

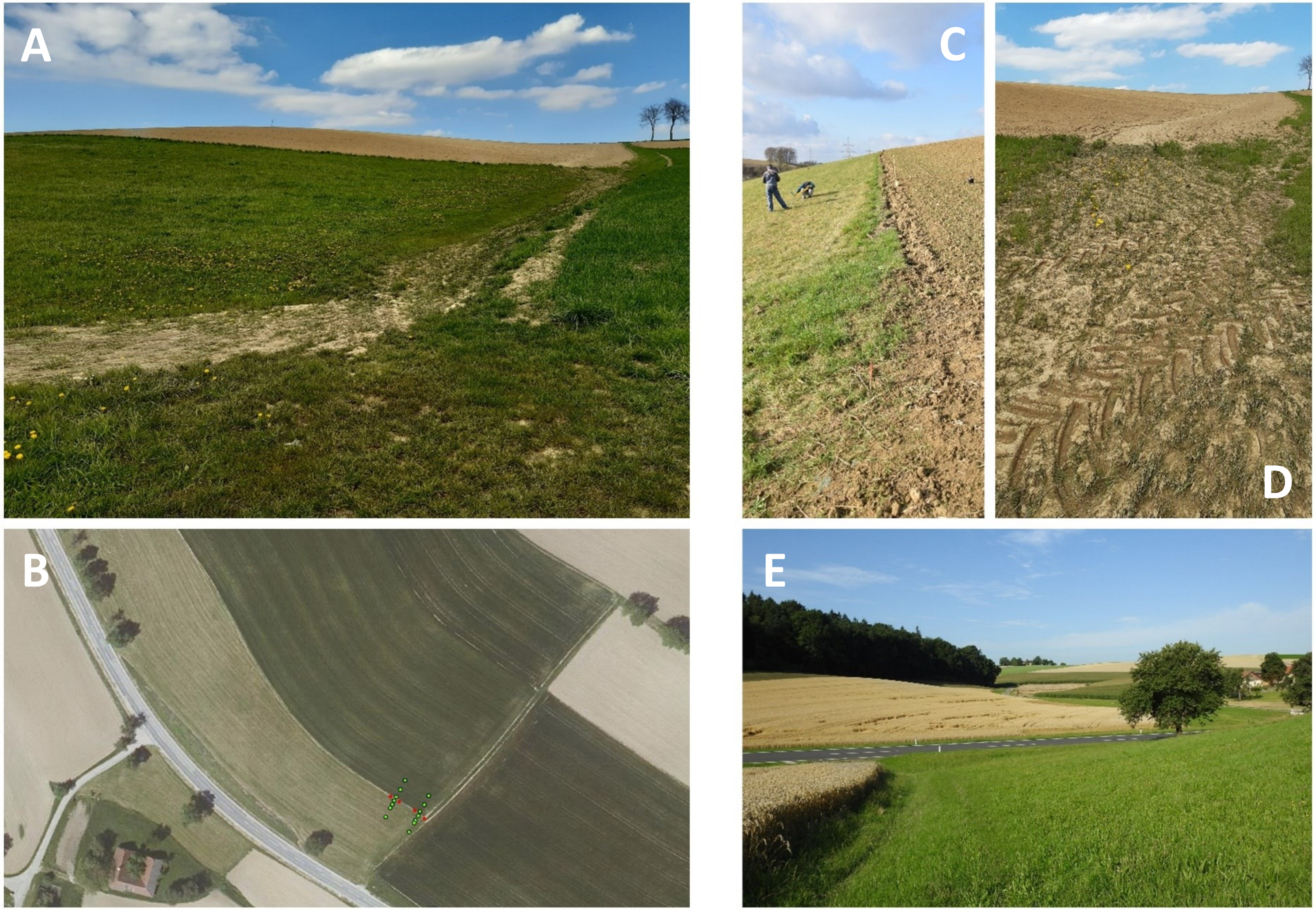


**Fig. A5** Site ME3. [A] Situation after a heavy rainfall event (May 2021). [B] Orthophoto of the VFS and the contributing agricultural area (2017). Green dots indicate the sampling points; red dots the actual field/VFS border. [C] Detailed view of the field/VFS transition (November 2021). [D] Close up of deposited sediment (May 2021). [E] View from the field edge into the VFS (July 2021).

Site *ME4*

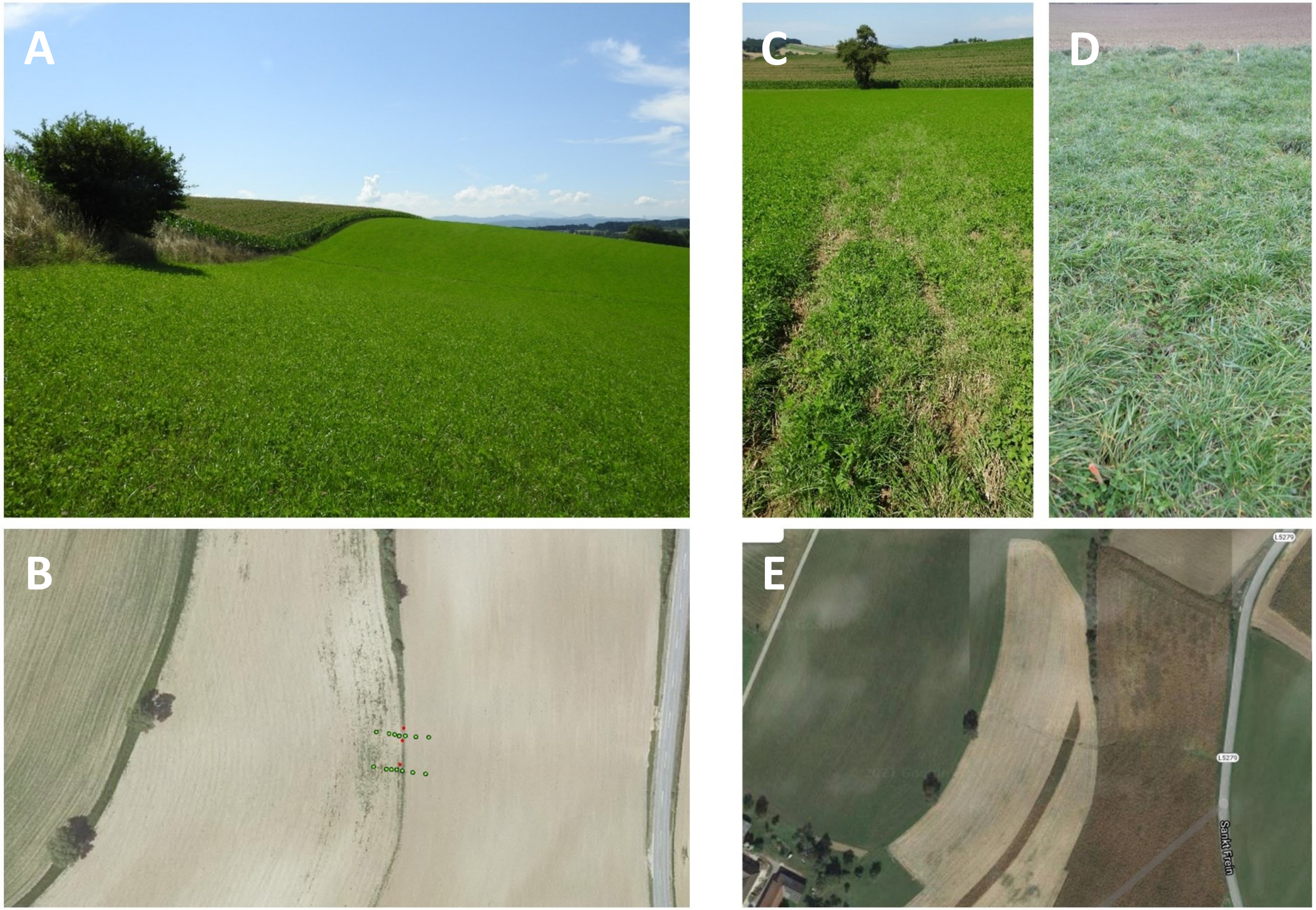


**Fig. A6** Site ME4. [A] Situation after a heavy rainfall event (July 2021). [B] Orthophoto of the VFS and the contributing agricultural area (2017). Green dots indicate the sampling points; red dots the actual field/VFS border. [C] Detailed view from the field edge into the VFS showing the erosion and runoff pathway (July 2021). [D] Detailed view from the VFS into the field showing the runoff pathway (November 2021). [E] Orthophoto of site ME4 with visible erosion pathway (October 2019; © Google Earth). Note, that the VFS used as an agricultural field until approximately one year before sampling.

**Table A1:** Crop rotation in the fields above the VFSs contributing to the flow accumulation (i.e., sources of sediment and nutrients). Green – grassland (in brackets the minimum number of cuts); red – erosion-prone crops; yellow – cereals; blue – other crops. The blue arrow indicates the direction of flow accumulation.

| **SB** | **2021** | **2020** | **2019** | **2018** | **2017** | **2016** | **2015** |
|---|---|---|---|---|---|---|---|
| *Field A2* | barley | maize | maize | barley | maize | barley | maize |
| *Field A1* | maize | maize | barley | maize | barley | maize | barley |
| *VFS* | grass (3) | grass (3) | grass (3) | grass (3) | grass (3) | grass (3) | grass (3) |

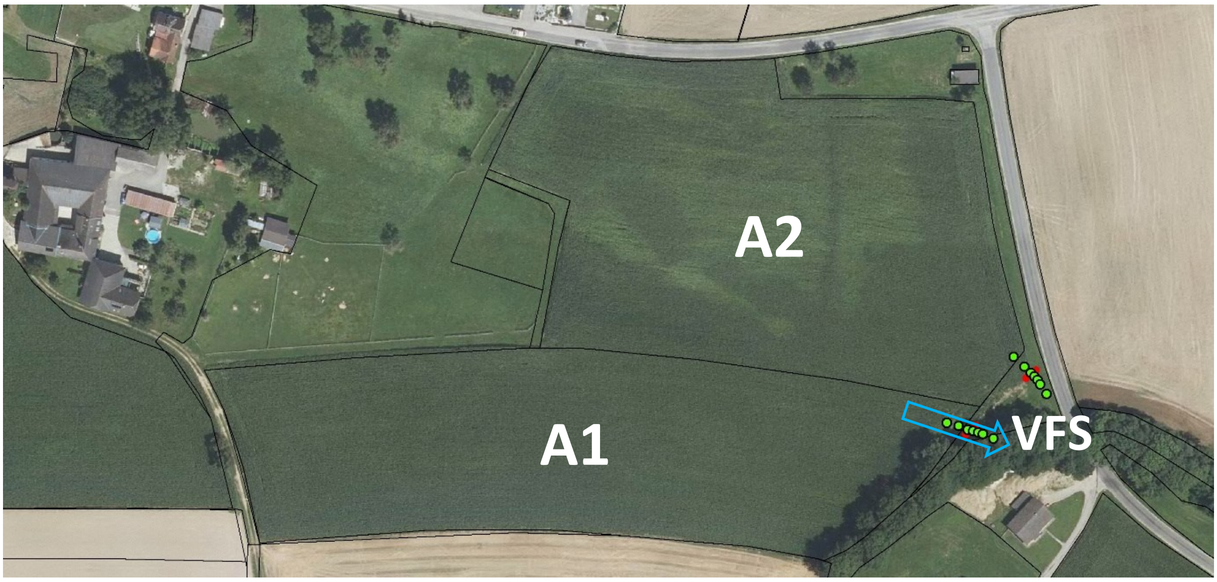


| **AM** | **2021** | **2020** | **2019** | **2018** | **2017** | **2016** | **2015** |
|---|---|---|---|---|---|---|---|
| *Field* | maize | barley | maize | maize | maize | wheat | maize |
| *VFS* | fodder | fodder | fodder | fodder | fodder | fodder | fodder |

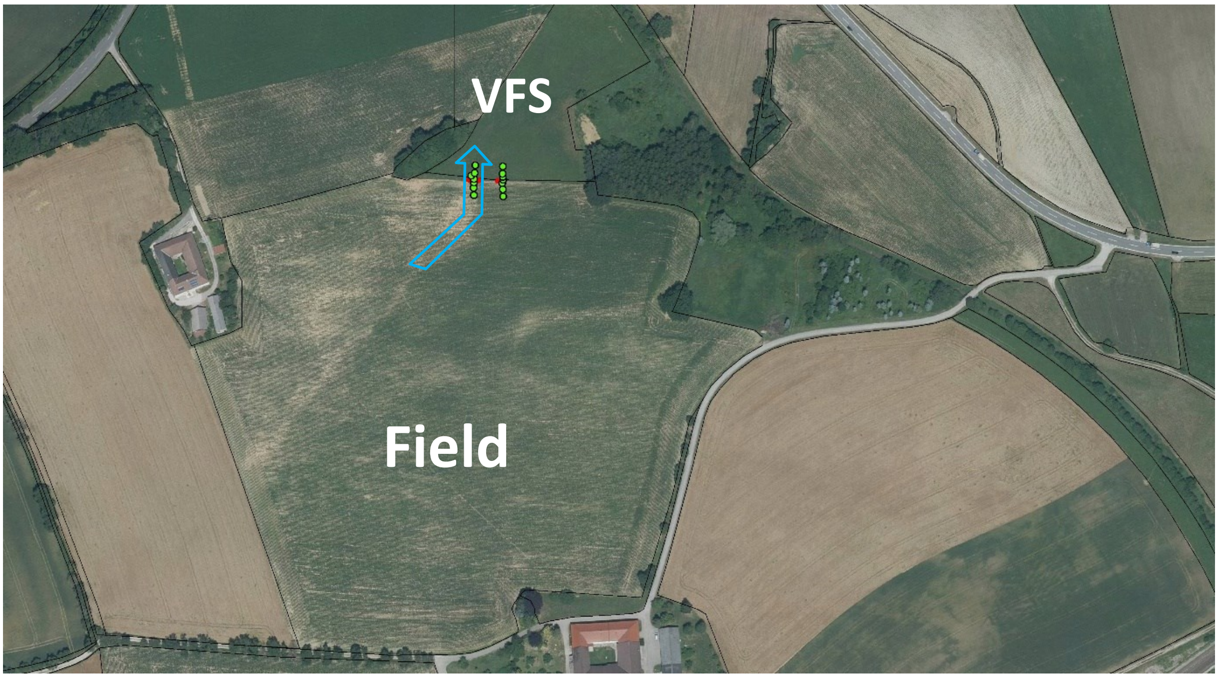

| HO | 2021 | 2020 | 2019 | 2018 | 2017 | 2016 | 2015 |
|---|---|---|---|---|---|---|---|
| *Field B3* | fallow | fallow | fallow | fallow | fallow | fallow | fallow |
| *Field B2* | barley | wheat | rape | barley | wheat | rape | barley |
| *Field B1* | barley | maize | triticale | rape | triticale | pumpkin | barley |
| *Field A* | maize | wheat | maize | wheat | maize | wheat | pumpkin |
| *VFS* | fallow | fallow | fallow | fallow | fallow | fallow | fallow |

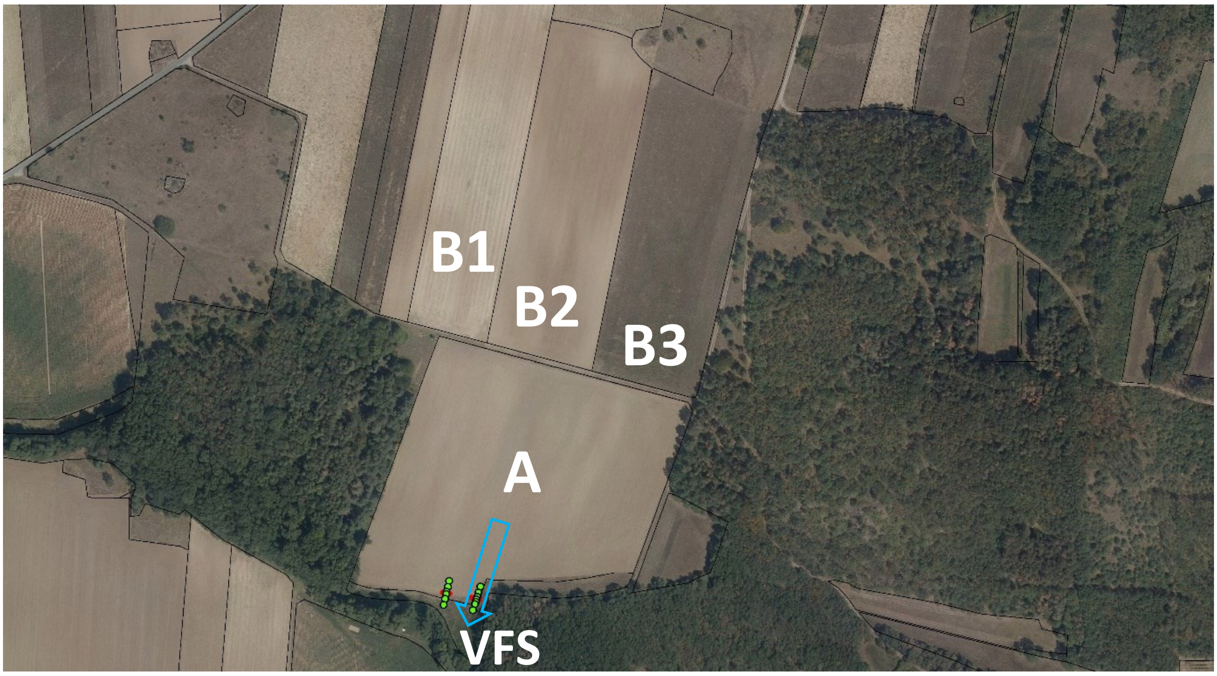


| PL | 2021 | 2020 | 2019 | 2018 | 2017 | 2016 | 2015 |
|---|---|---|---|---|---|---|---|
| *Meadow* | grass (3) | grass (3) | grass (3) | grass (3) | grass (3) | grass (3) | grass (3) |
| *Field A* | barley | maize | maize | maize | barley | maize | maize |
| *VFS* | grass (3) | grass (3) | grass (3) | grass (3) | grass (3) | grass (3) | grass (3) |

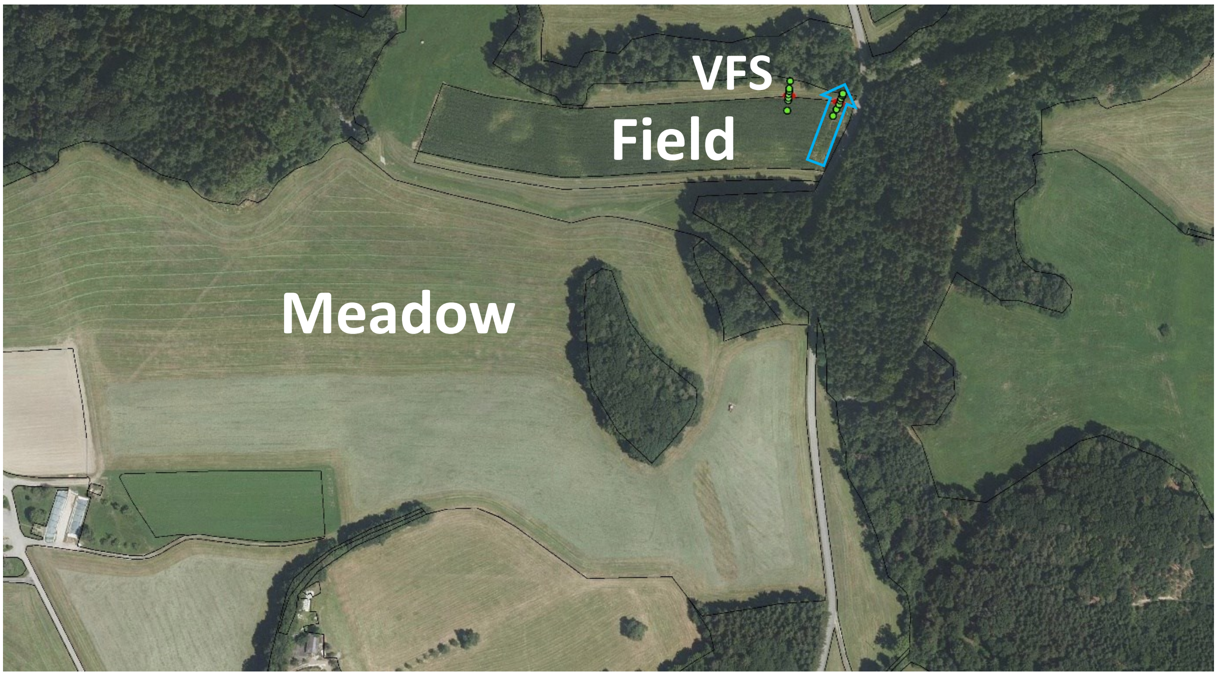

| **ME3** | **2021** | **2020** | **2019** | **2018** | **2017** | **2016** | **2015** |
|---|---|---|---|---|---|---|---|
| *Field B* | soy | barley | wheat | soy | wheat | maize | clover grass |
| *Field A* | soy | barley | wheat | soy | barley | wheat | maize |
| *VFS* | grass (3) | grass (3) | grass (3) | grass (3) | grass (3) | grass (3) | grass (3) |

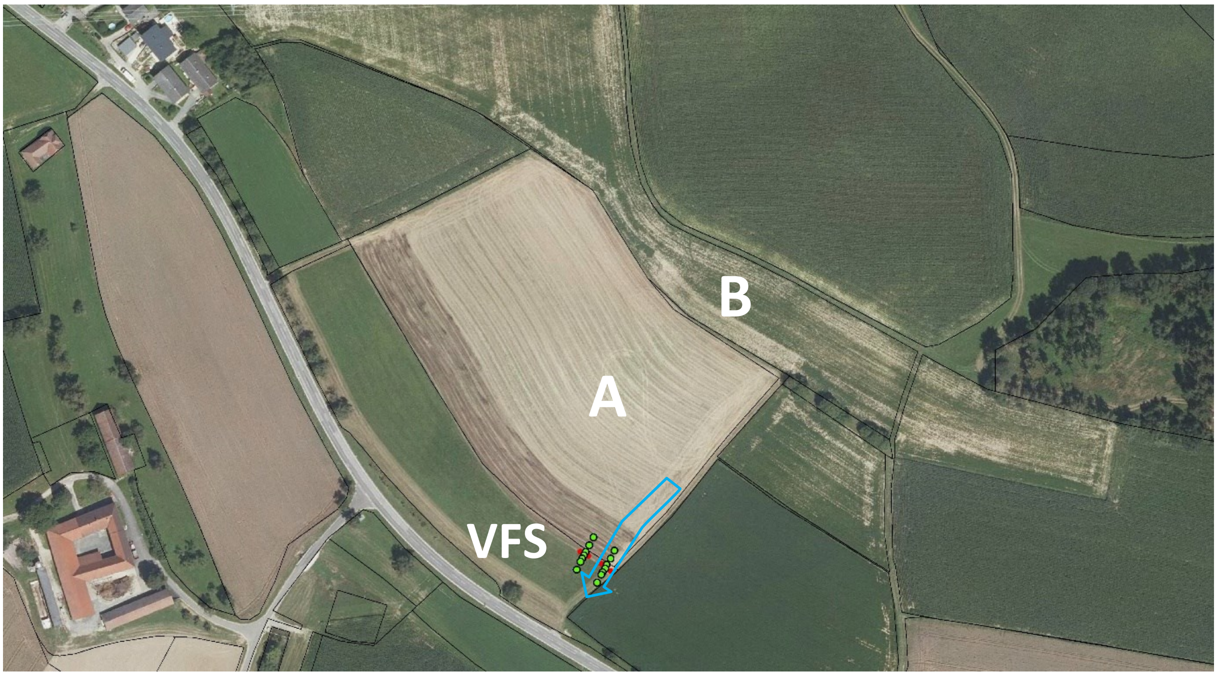


| **ME4** | **2021** | **2020** | **2019** | **2018** | **2017** | **2016** | **2015** |
|---|---|---|---|---|---|---|---|
| *Field* | maize | wheat | maize | wheat | maize | wheat | maize |
| *VFS* | fodder | wheat | maize | soy | caraway | wheat | caraway |

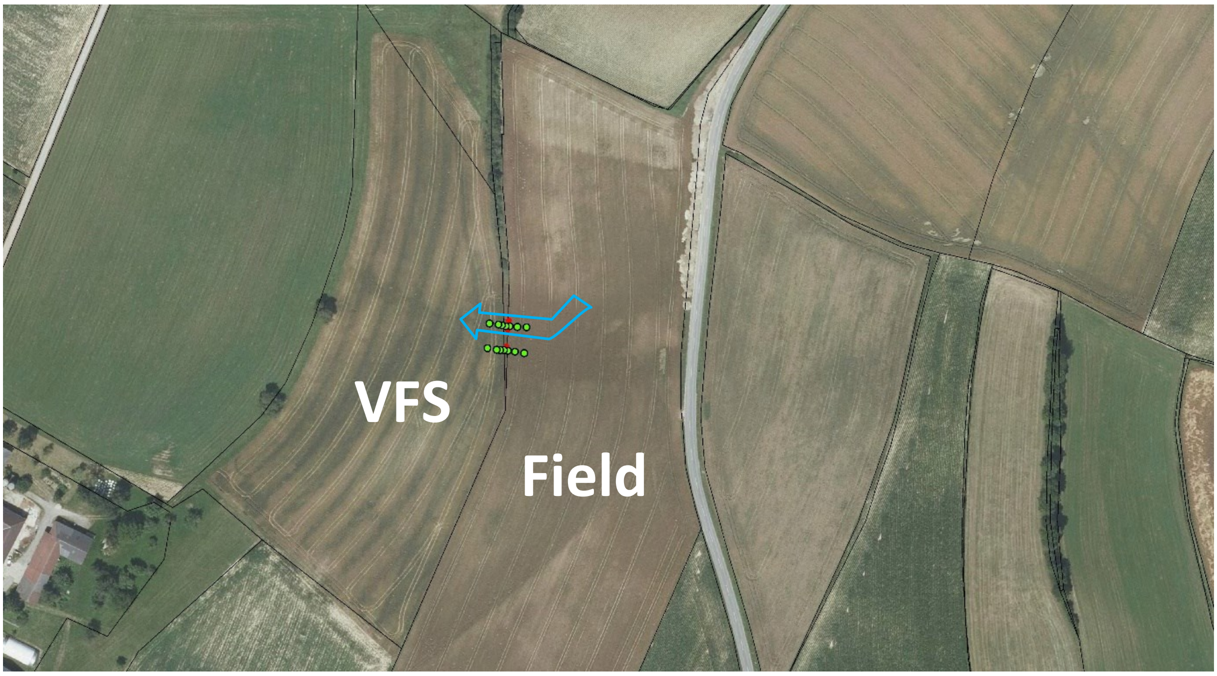

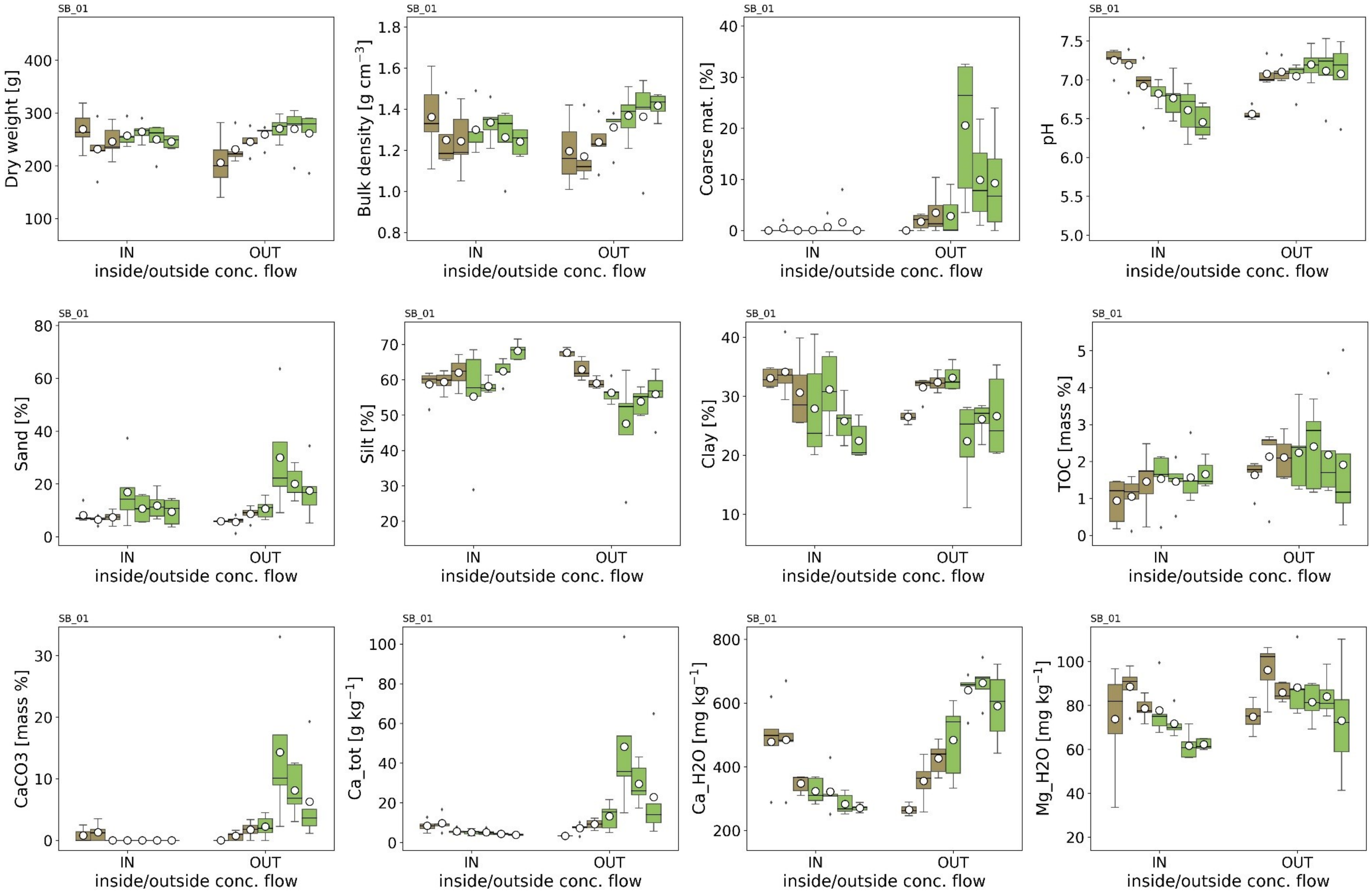


**Fig. B1** Site SB. Distribution and concentration of all analyzed physical and chemical soil parameters. Boxplots integrate over all depth classes. Brown – Field, Green – VFS. White dots indicate the mean, black lines the median, the boxes the 25 and 75-percentiles, the whiskers the 5 and 95-percentiles, and the diamonds outliers. For details about parameters see the main text and Ramler & Strauss (2023).

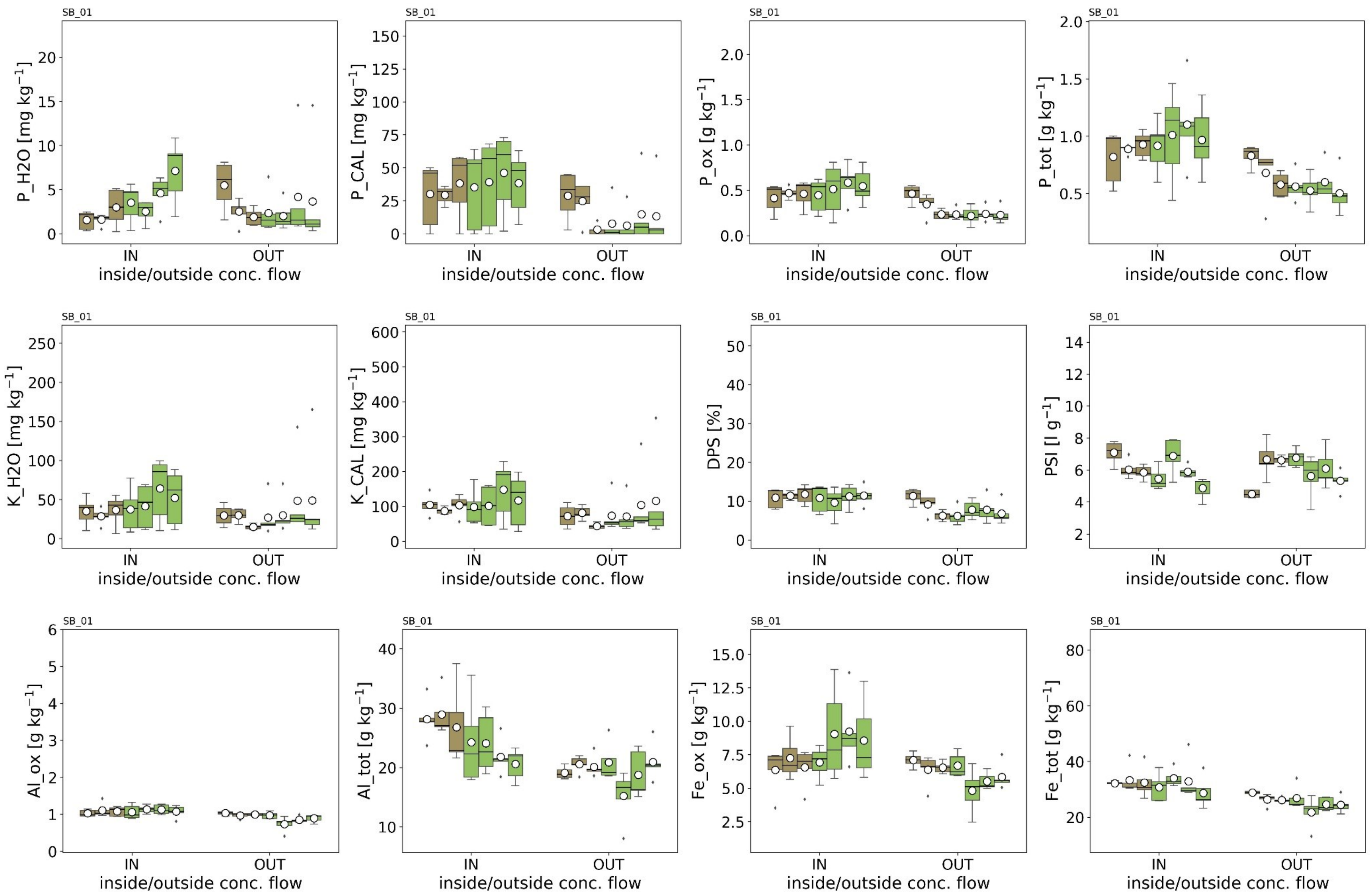


**Fig. B2** Site SB (continued).

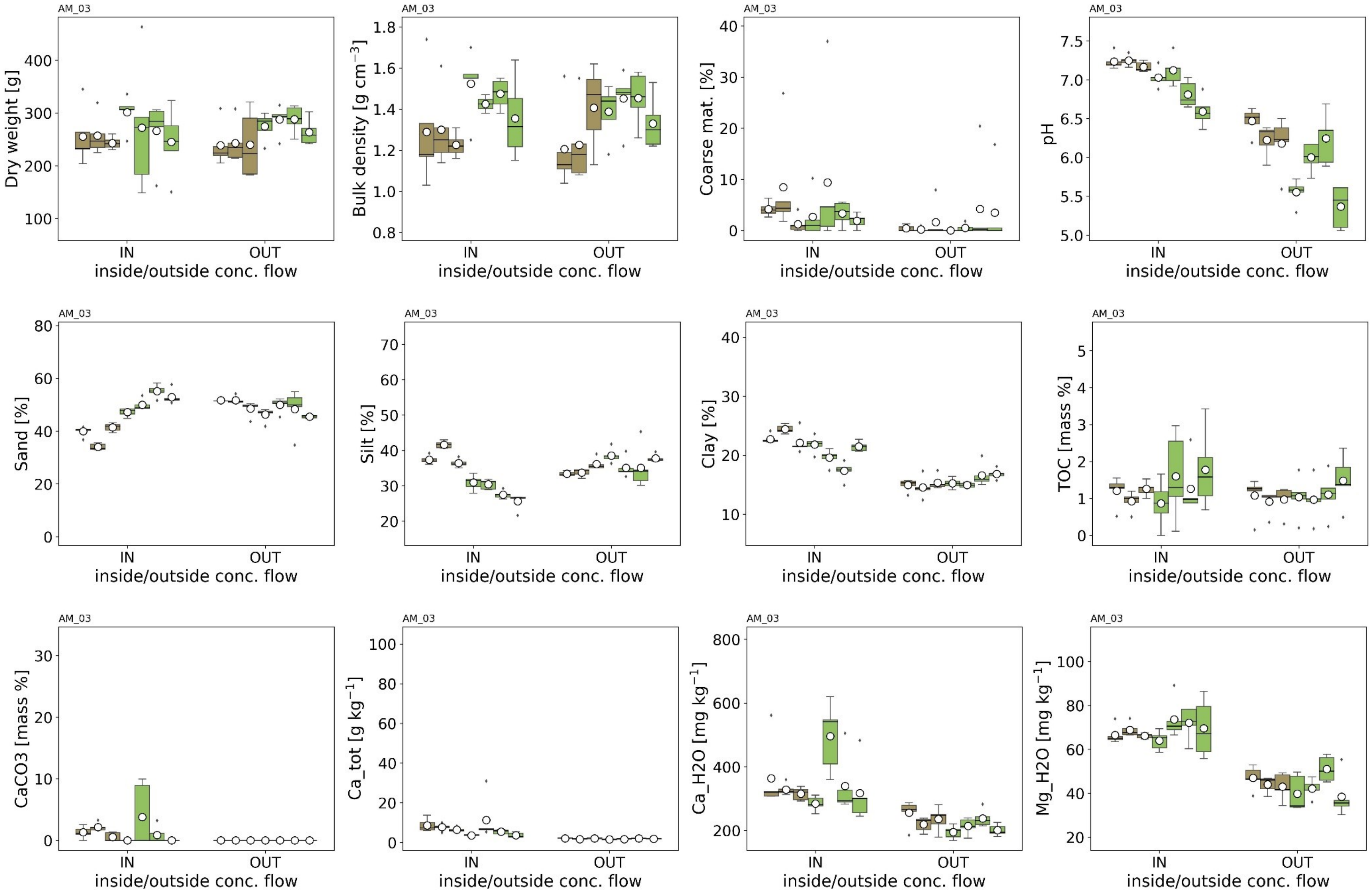


**Fig. C1** Site AM. Distribution and concentration of all analyzed physical and chemical soil parameters. Boxplots integrate over all depth classes. Brown – Field, Green – VFS. White dots indicate the mean, black lines the median, the boxes the 25 and 75-percentiles, the whiskers the 5 and 95-percentiles, and the diamonds outliers. For details about parameters see the main text and Ramler & Strauss (2023).

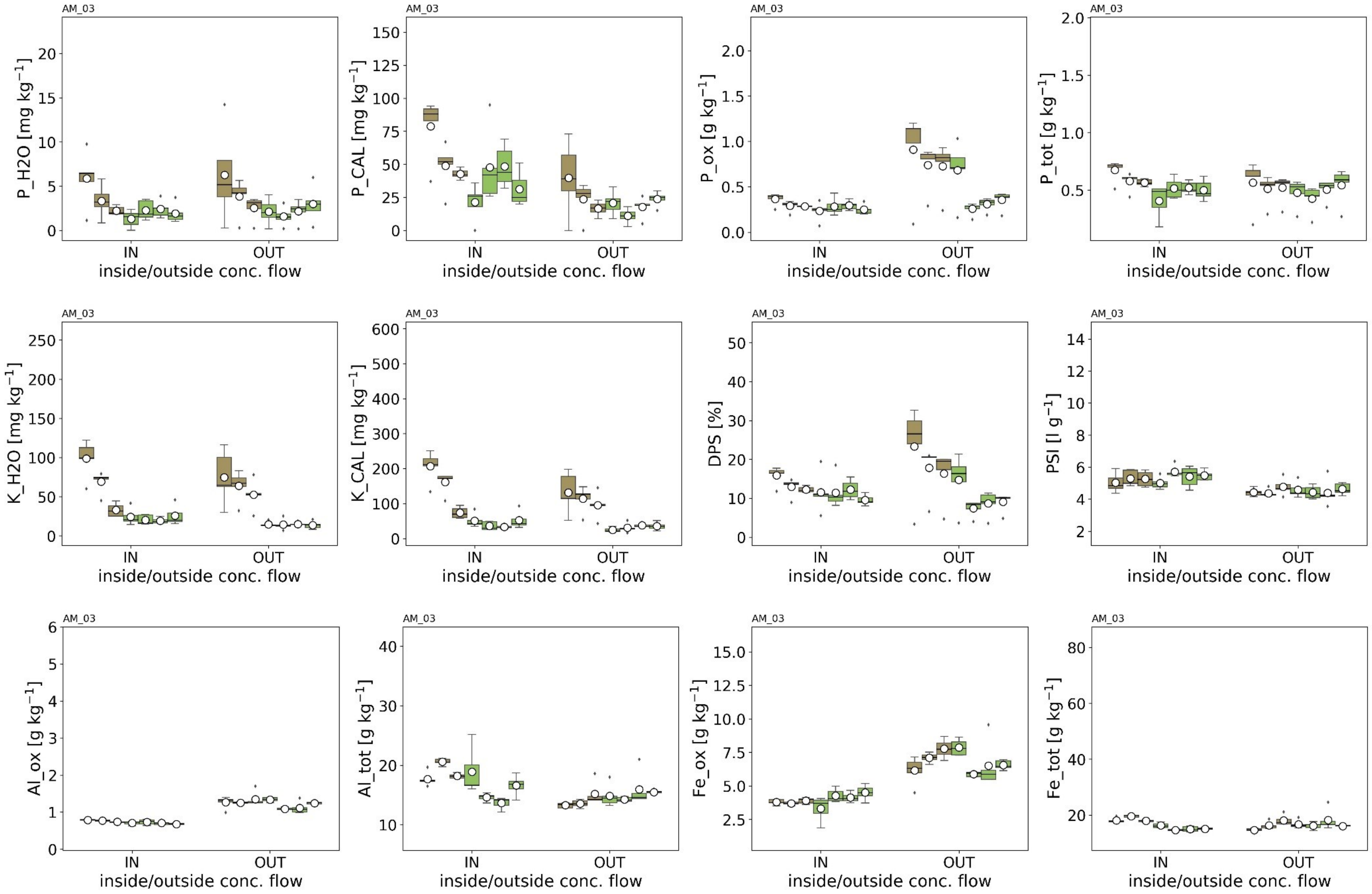


**Fig. C2** Site AM (continued).

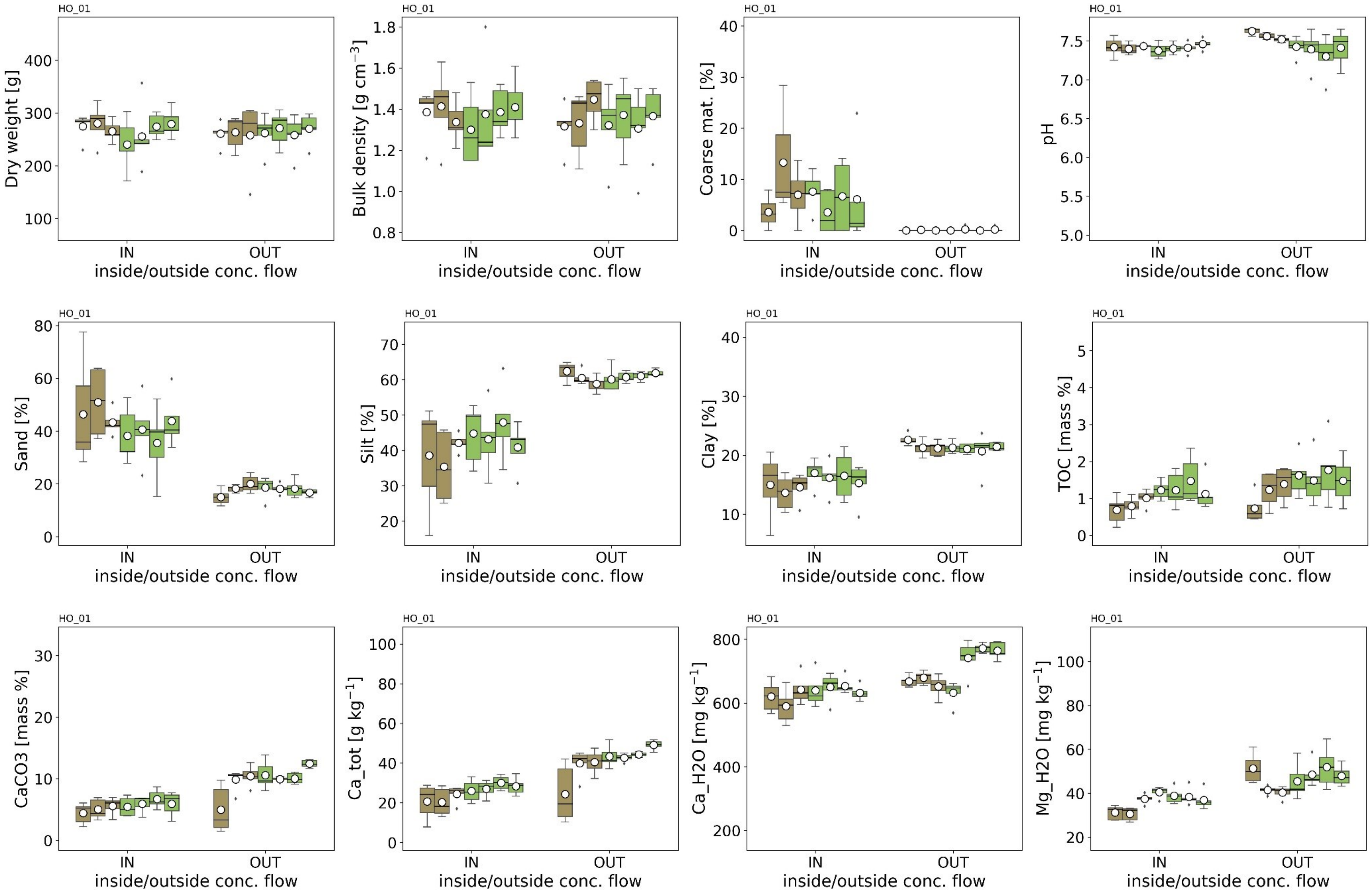


**Fig. D1** Site HO. Distribution and concentration of all analyzed physical and chemical soil parameters. Boxplots integrate over all depth classes. Brown – Field, Green – VFS. White dots indicate the mean, black lines the median, the boxes the 25 and 75-percentiles, the whiskers the 5 and 95-percentiles, and the diamonds outliers. For details about parameters see the main text and Ramler & Strauss (2023).

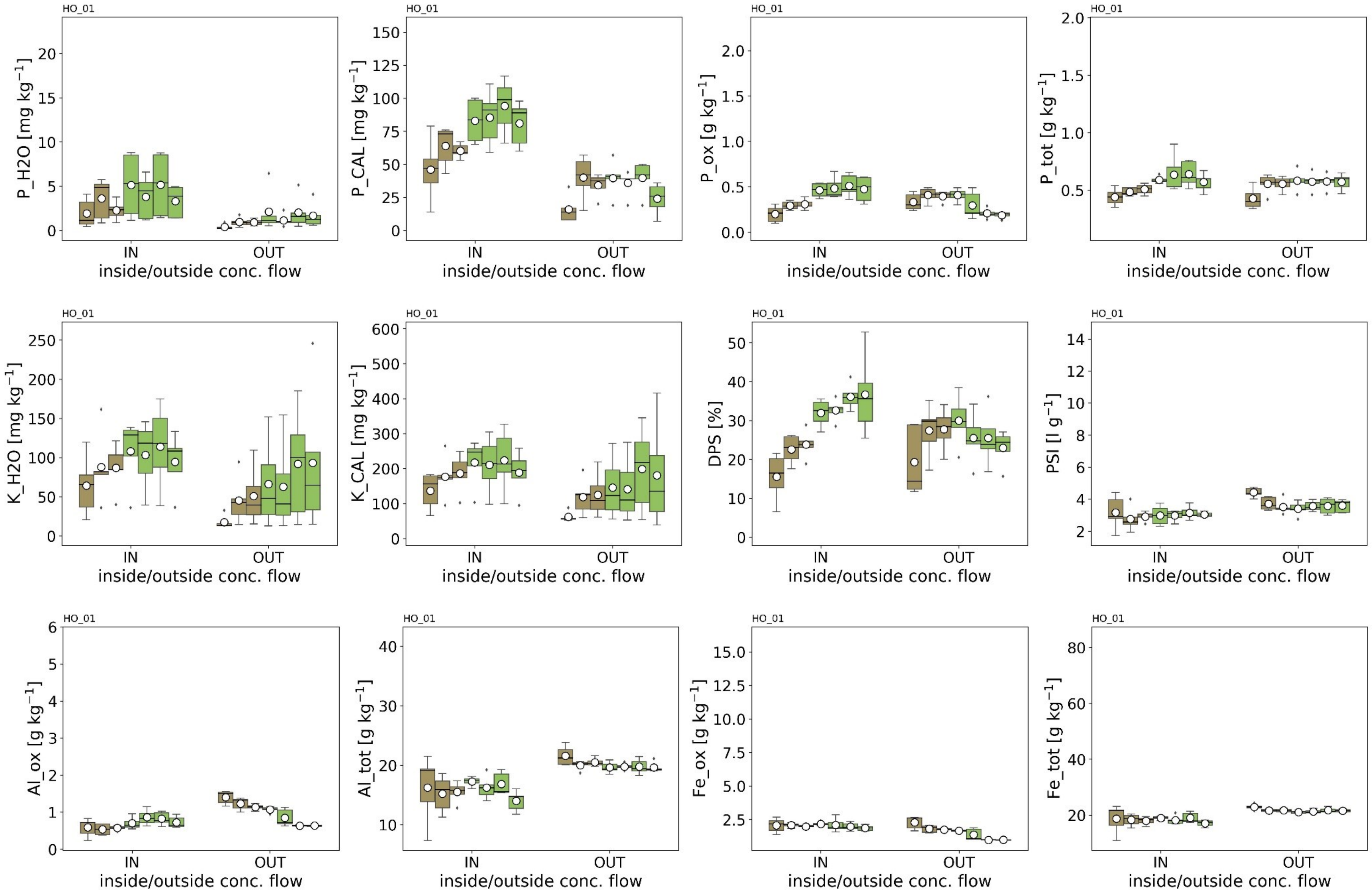


**Fig. D2** Site HO (continued).

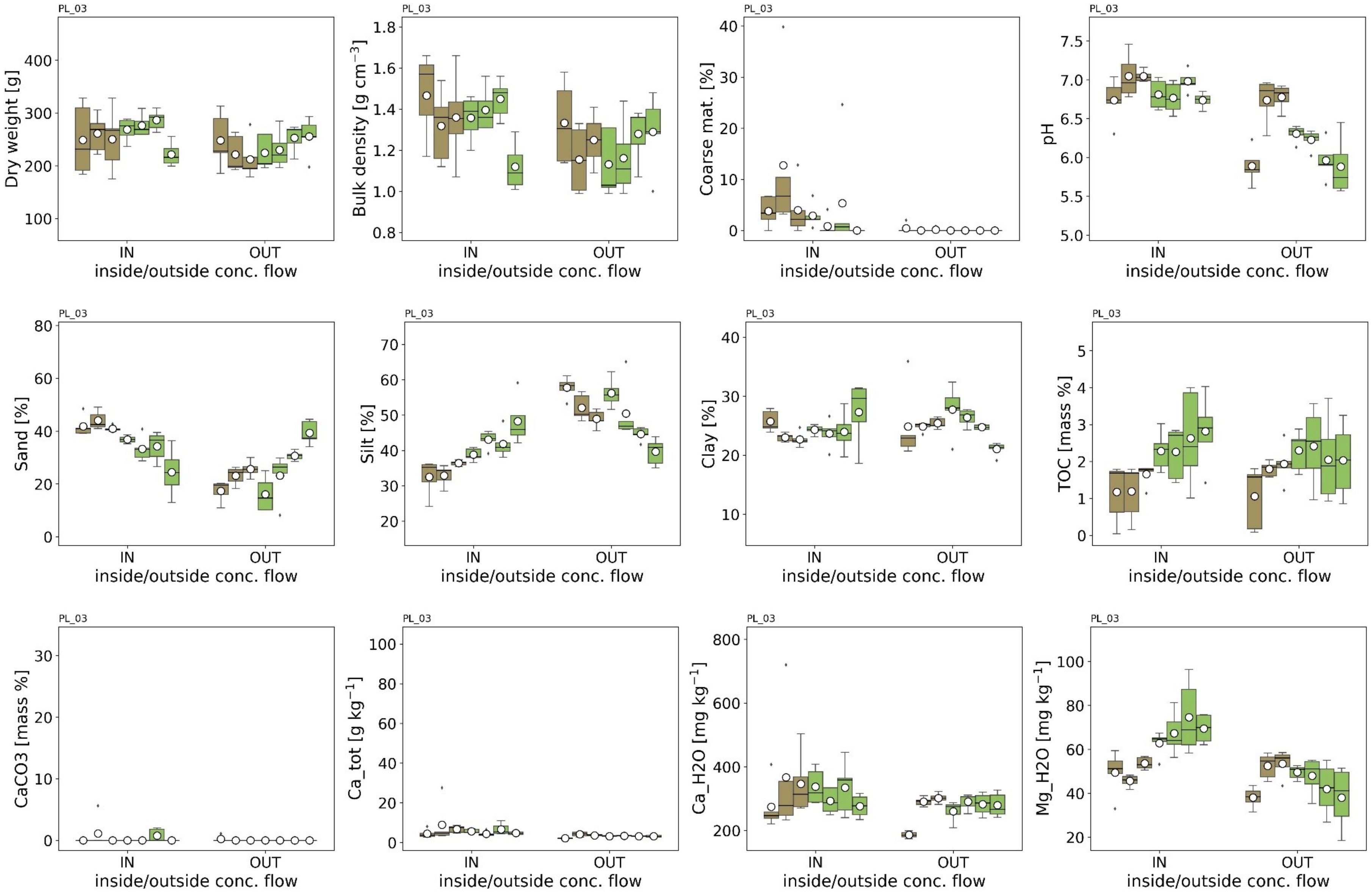


**Fig. E1** Site PL. Distribution and concentration of all analyzed physical and chemical soil parameters. Boxplots integrate over all depth classes. Brown – Field, Green – VFS. White dots indicate the mean, black lines the median, the boxes the 25 and 75-percentiles, the whiskers the 5 and 95-percentiles, and the diamonds outliers. For details about parameters see the main text and Ramler & Strauss (2023).

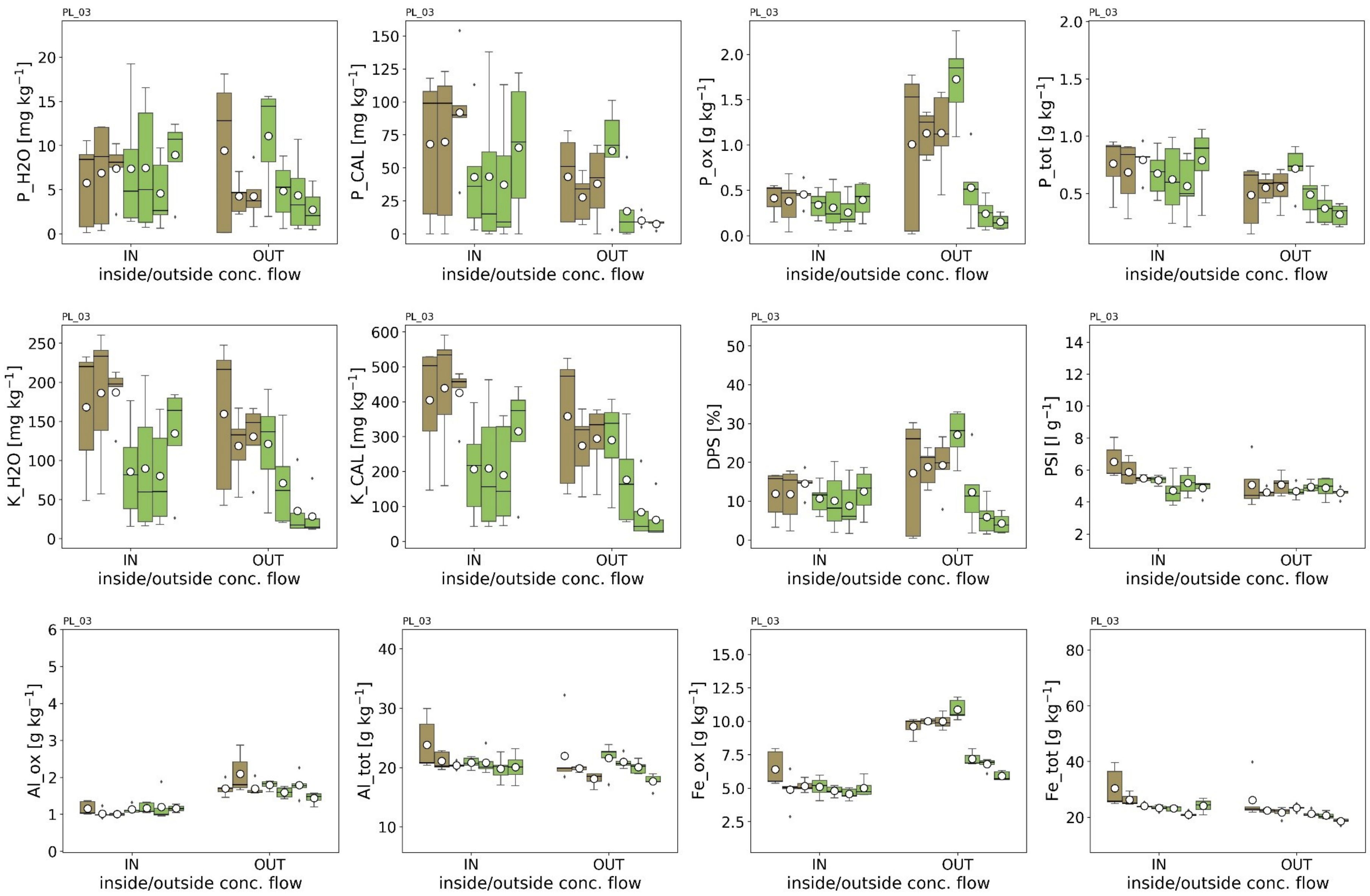


**Fig. E2** Site PL (continued).

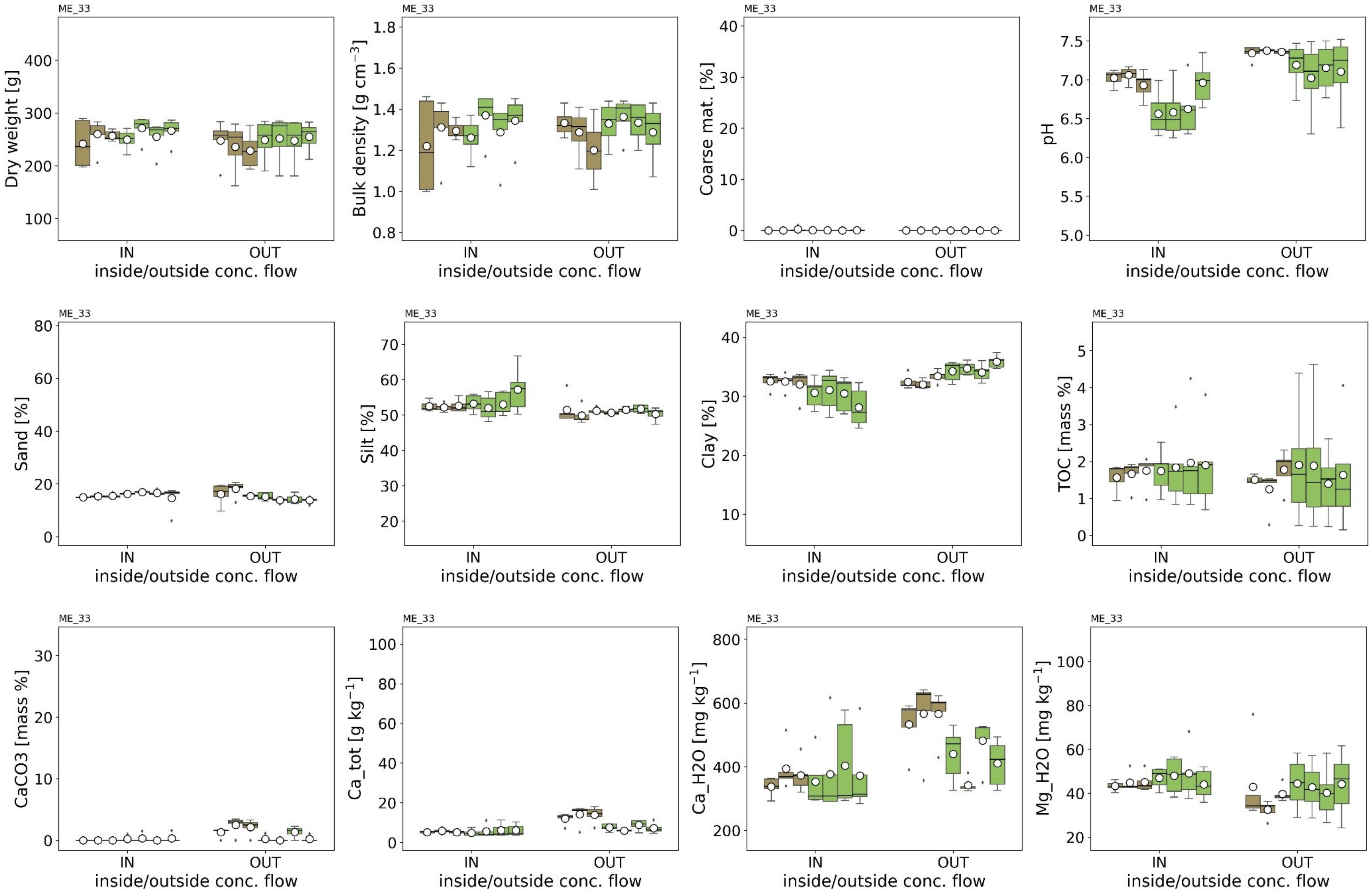


**Fig. F1** Site ME3. Distribution and concentration of all analyzed physical and chemical soil parameters. Boxplots integrate over all depth classes. Brown – Field, Green – VFS. White dots indicate the mean, black lines the median, the boxes the 25 and 75-percentiles, the whiskers the 5 and 95-percentiles, and the diamonds outliers. For details about parameters see the main text and Ramler & Strauss (2023).

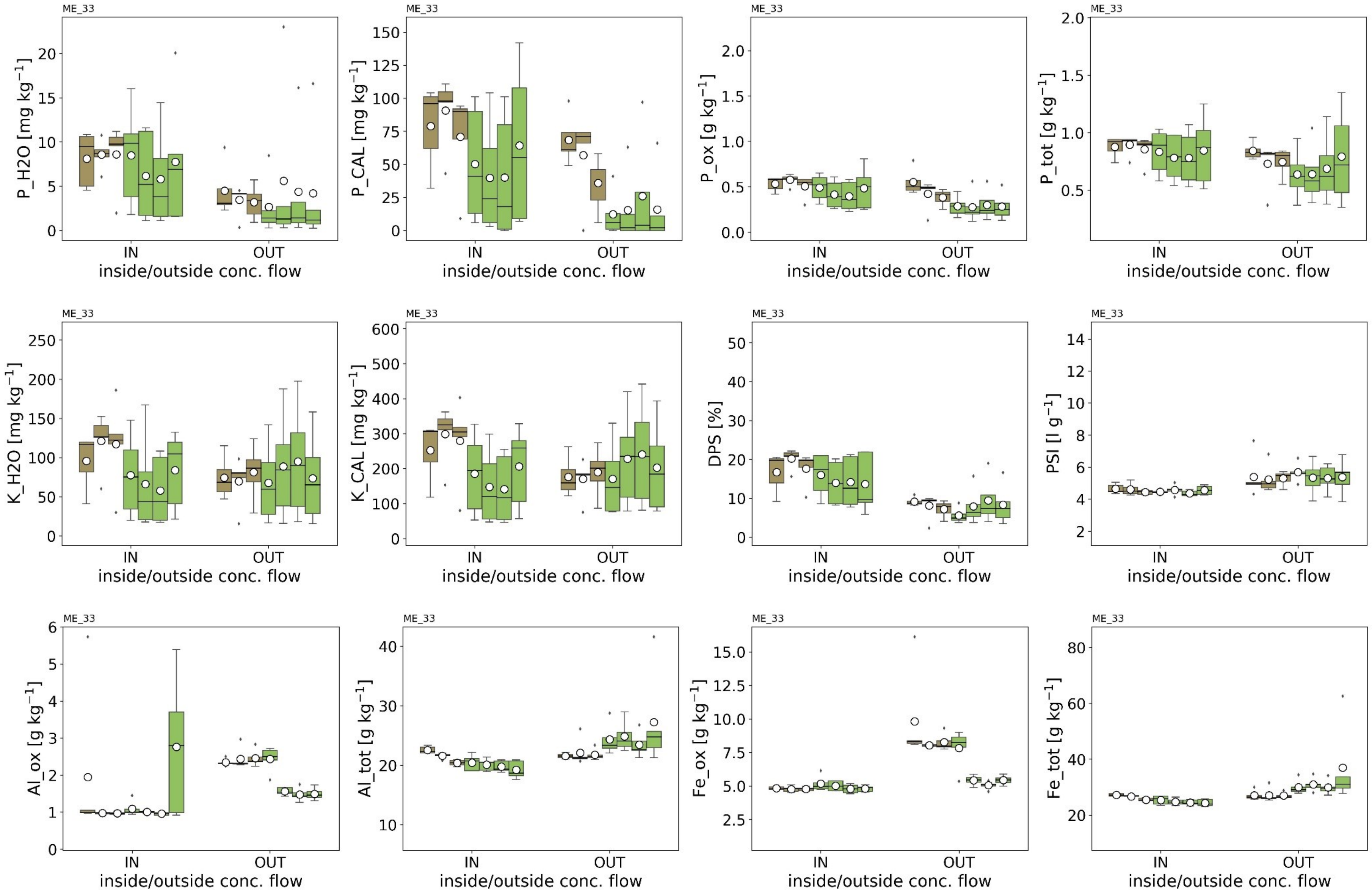


**Fig. F2** Site ME3 (continued).

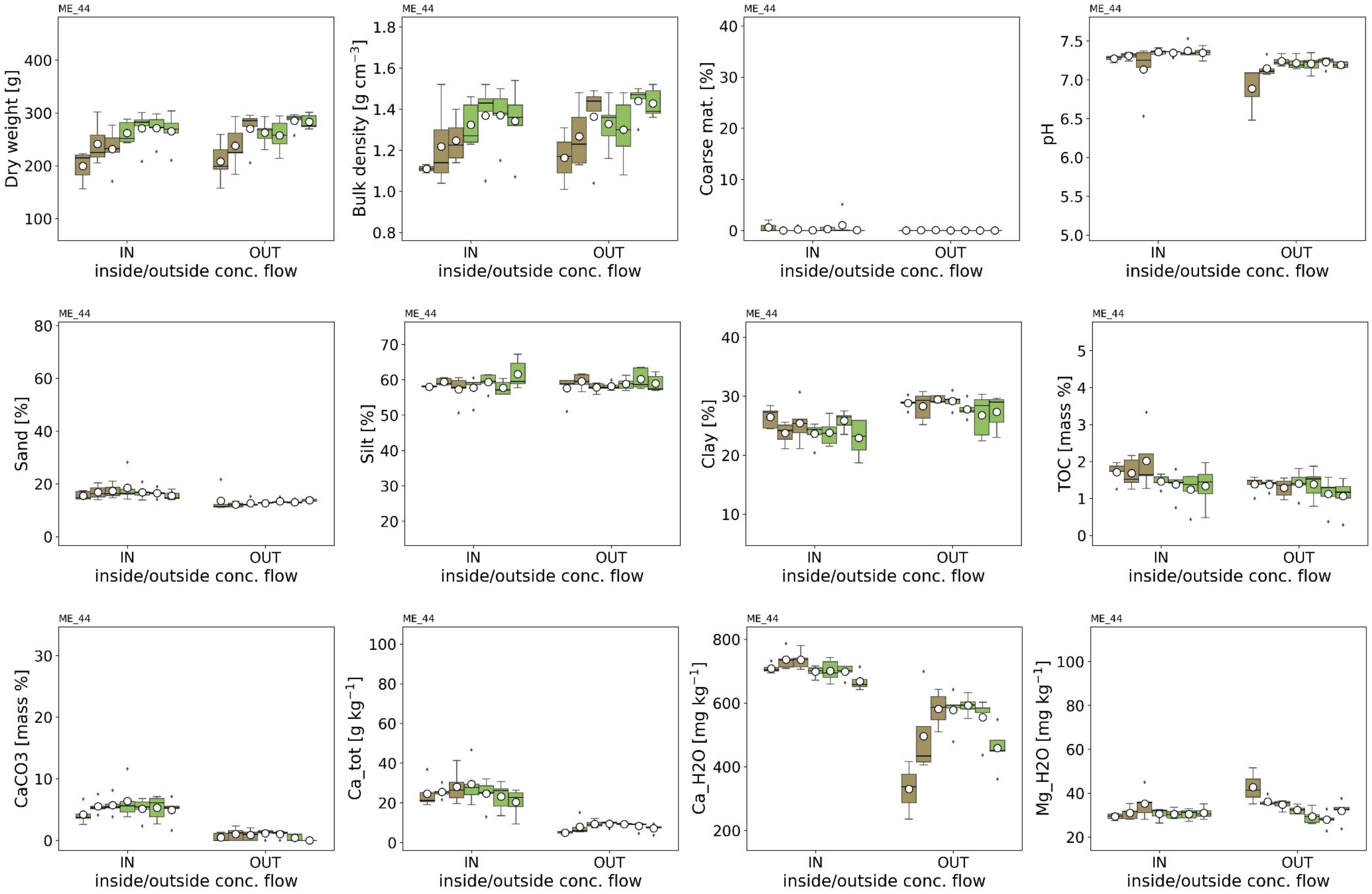


**Fig. G1** Site ME4. Distribution and concentration of all analyzed physical and chemical soil parameters. Boxplots integrate over all depth classes. Brown – Field, Green – VFS. White dots indicate the mean, black lines the median, the boxes the 25 and 75-percentiles, the whiskers the 5 and 95-percentiles, and the diamonds outliers. For details about parameters see the main text and Ramler & Strauss (2023).

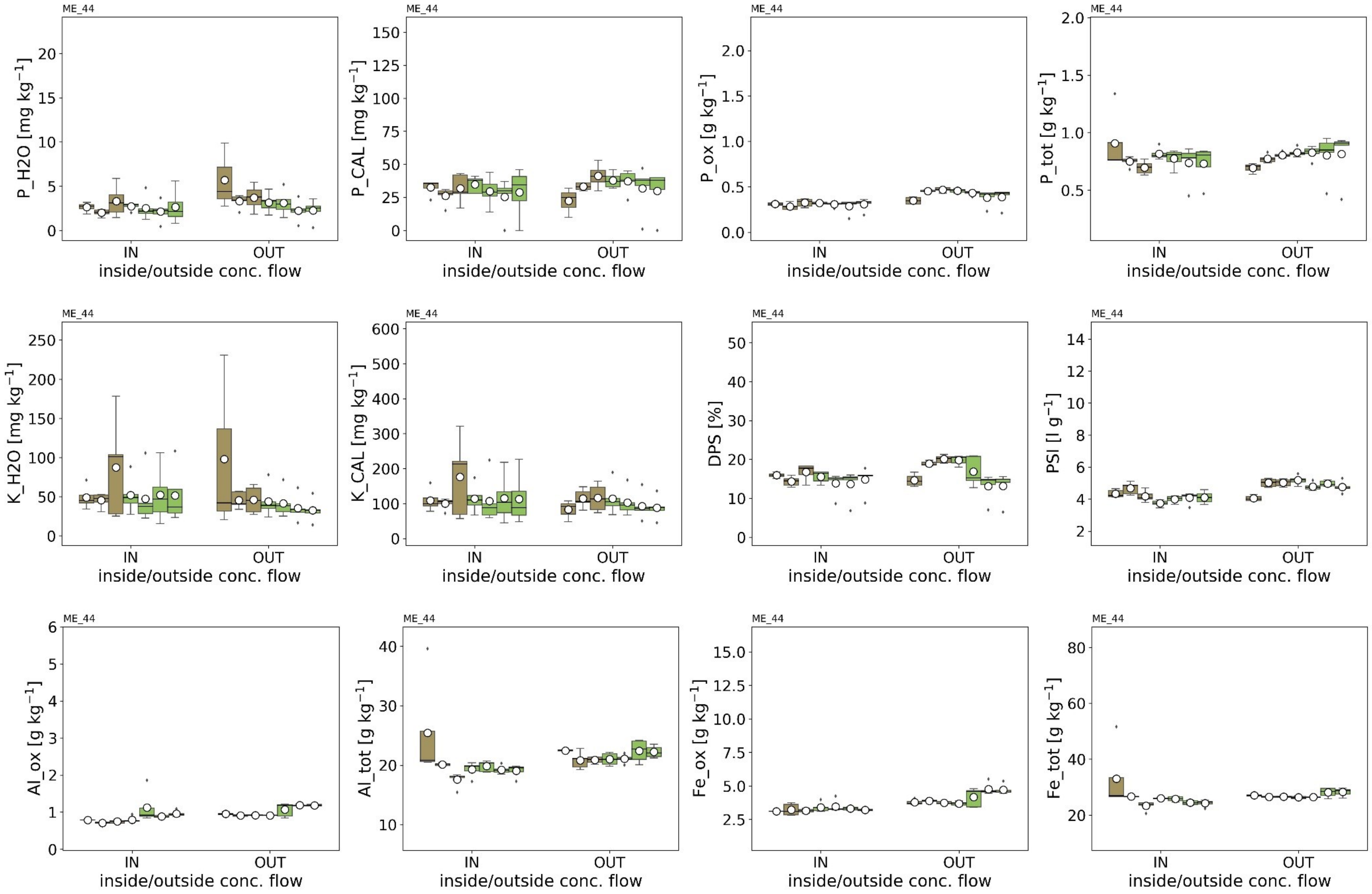


**Fig. G2** Site ME4 (continued).
